# Modeling Skeletal Muscle Laminopathies Using Human Induced Pluripotent Stem Cells Carrying Pathogenic *LMNA* Mutations

**DOI:** 10.3389/fphys.2018.01332

**Published:** 2018-10-15

**Authors:** Heather B. Steele-Stallard, Luca Pinton, Shilpita Sarcar, Tanel Ozdemir, Sara M. Maffioletti, Peter S. Zammit, Francesco Saverio Tedesco

**Affiliations:** ^1^Department of Cell and Developmental Biology, University College London, London, United Kingdom; ^2^Randall Centre for Cell and Molecular Biophysics, King's College London, London, United Kingdom; ^3^The Dubowitz Neuromuscular Centre, UCL Great Ormond Street Institute of Child Health, London, United Kingdom

**Keywords:** lamin A/C, *LMNA*, skeletal muscle, laminopathies, muscular dystrophy, iPSCs, disease modeling, 3D modeling

## Abstract

Laminopathies are a clinically heterogeneous group of disorders caused by mutations in *LMNA*. The main proteins encoded by *LMNA* are Lamin A and C, which together with Lamin B1 and B2, form the nuclear lamina: a mesh-like structure located underneath the inner nuclear membrane. Laminopathies show striking tissue specificity, with subtypes affecting striated muscle, peripheral nerve, and adipose tissue, while others cause multisystem disease with accelerated aging. Although several pathogenic mechanisms have been proposed, the exact pathophysiology of laminopathies remains unclear, compounded by the rarity of these disorders and lack of easily accessible cell types to study. To overcome this limitation, we used induced pluripotent stem cells (iPSCs) from patients with skeletal muscle laminopathies such as *LMNA*-related congenital muscular dystrophy and limb-girdle muscular dystrophy 1B, to model disease phenotypes *in vitro*. iPSCs can be derived from readily accessible cell types, have unlimited proliferation potential and can be differentiated into cell types that would otherwise be difficult and invasive to obtain. iPSC lines from three skeletal muscle laminopathy patients were differentiated into inducible myogenic cells and myotubes. Disease-associated phenotypes were observed in these cells, including abnormal nuclear shape and mislocalization of nuclear lamina proteins. Nuclear abnormalities were less pronounced in monolayer cultures of terminally differentiated skeletal myotubes than in proliferating myogenic cells. Notably, skeletal myogenic differentiation of *LMNA*-mutant iPSCs in artificial muscle constructs improved detection of myonuclear abnormalities compared to conventional monolayer cultures across multiple pathogenic genotypes, providing a high-fidelity modeling platform for skeletal muscle laminopathies. Our results lay the foundation for future iPSC-based therapy development and screening platforms for skeletal muscle laminopathies.

## Introduction

The *LMNA* gene encodes two major protein isoforms: Lamin A and C; these nuclear intermediate filament proteins are expressed in most somatic cells, but absent from undifferentiated cells such as embryonic, germ and pluripotent cells (Dechat et al., 2010a; Worman, 2012). At the nuclear periphery, Lamin A/C, together with Lamin B1 and B2, forms the nuclear lamina, a protein meshwork that underlies the nuclear membrane. The nuclear lamina provides structural support to the nucleus, and participates in mechanotransduction, heterochromatin tethering and regulation of transcription (Azibani et al., 2014; Gruenbaum and Foisner, 2015). Lamin A/C is also present in the nucleoplasm, where it is thought to be involved in regulation of cell proliferation, differentiation, chromatin organization and DNA replication (Dechat et al., 2010b).

Mutations in *LMNA* cause at least 16 rare disorders, collectively known as laminopathies (Scharner et al., 2010; Worman, 2012). Laminopathies demonstrate tissue-specific phenotypes and can be grouped into those affecting striated cardiac and/or skeletal muscle (the most common group), peripheral nerve or adipose tissue, and those causing multisystem disease with accelerated aging. Striated muscle laminopathies are usually caused by missense mutations, typically having a dominant inheritance and include dilated cardiomyopathy (DCM), Emery-Dreifuss muscular dystrophy (EDMD), limb-girdle muscular dystrophy type 1B (LGMD1B) and *LMNA*-related congenital muscular dystrophy (L-CMD). While all four have cardiac involvement, EDMD, LGMD1B and L-CMD also affect skeletal muscle (Worman, 2012; Bonne and Quijano-Roy, 2013; Azibani et al., 2014). However, genotype-phenotype correlations are not always clear, with instances of the same *LMNA* mutation causing different disorders (Scharner et al., 2010, 2014; Bertrand et al., 2011). Two non-mutually exclusive theories have been proposed to explain the pathological causes of striated muscle laminopathies. In the mechanical stress hypothesis, mutations in Lamin A/C lead to a nucleus that is more vulnerable to damage from mechanical force during muscle contraction. The gene expression and stem cell differentiation hypothesis suggests that mutant Lamin A/C deregulates expression of certain genes, which causes defective cell differentiation and function (Azibani et al., 2014; Gruenbaum and Foisner, 2015).

A typical cellular hallmark of *LMNA* mutations is abnormal nuclear morphology, as observed in muscle biopsies of EDMD patients (Park et al., 2009). Such nuclear abnormalities have been modeled *in vitro* in primary fibroblasts and C2C12 myoblasts ectopically expressing pathogenic *LMNA* mutations. Fibroblasts from patients with LGMD1B (Muchir et al., 2003), autosomal dominant EDMD (Muchir et al., 2004), L-CMD (Tan et al., 2015), DCM (Muchir et al., 2004), familial partial lipodystrophy (FPLD) (Vigouroux et al., 2001; Verstraeten et al., 2009), Mandibuloacral dysplasia (MAD) (Novelli et al., 2002), Hutchinson-Gilford progeria syndrome (HGPS) (Eriksson et al., 2003), and Werner syndrome 2 (WRN2) (Chen et al., 2003) all have nuclear abnormalities, such as abnormal nuclear shape and mislocalization of lamina proteins. These can be characterized by: (1) mislocalization of Lamin A/C protein (e.g., into structures with a honeycomb-like appearance), (2) nucleoplasmic foci and/or increased nucleoplasmic/lamina ratios, (3) areas with no lamin B1 (capping), and (4) Emerin mislocalization. C2C12 mouse myoblasts expressing EDMD/L-CMD-causing *LMNA* mutations exhibit similar defects (Ostlund et al., 2001; Favreau et al., 2003; Markiewicz et al., 2005; Scharner et al., 2011; Barateau et al., 2017). However, not all *LMNA-*mutations produce a morphological defect or have Lamin A/C mislocalization (Ostlund et al., 2001; Muchir et al., 2004), which can depend on the cell type assessed (e.g., patient fibroblasts vs. over-expression in C2C12 myoblasts) (Barateau et al., 2017). Furthermore, correlations between a particular laminopathy and the type of nuclear shape and/or mislocalization defects have remained elusive, with examples of different tissue specific disorders causing the same type of mislocalizations and shape defects (Vigouroux et al., 2001; Novelli et al., 2002; Muchir et al., 2004).

Understanding pathological events underlying laminopathies and making genotype-phenotype correlations has been limited by the rarity of these disorders, invasiveness of sampling and limited proliferation/differentiation capacity of primary patient cells. This has led to fibroblasts typically being studied as a primary cell source, despite not being the disease-causing/-affected cell type in most laminopathies. Induced pluripotent stem cells (iPSCs) (Takahashi et al., 2007) offer a new approach to disease modeling and drug screening (Ebert et al., 2012; Brandão et al., 2017). IPSCs can be made from patient primary cells obtained from minimally invasive sources, such as skin fibroblasts, blood or urine cells, which can then be reprogrammed to pluripotency and directed to differentiate into any cell type, enabling the study of disease-/patient-specific cells that would otherwise be difficult to obtain. iPSCs also possess a virtually unlimited expansion potential, circumventing problems of cellular senescence of primary cells. This is particularly important for laminopathies, where loss of disease-associated phenotypes can occur with prolonged culture (Muchir et al., 2004). An additional advantage of iPSC derivatives is that they contain endogenous levels of Lamin A/C, unlike traditional systems of ectopic overexpression of *LMNA* mutants in myoblasts; or *Lmna*-mutant mice that often require homozygous mutations to manifest a phenotype (e.g., Arimura et al., 2005), but even then phenotypes in mice can still be subtle (e.g., Poitelon et al., 2012).

Most studies have focused on iPSCs derived from patients with HGPS (Lo Cicero and Nissan, 2015), and to a lesser extent DCM, FPLD, and atypical Werners (Ho et al., 2011; Siu et al., 2012; Friesen and Cowan, 2017; Lee et al., 2017). Differentiated FPLD-iPSCs exhibit disease associated defects in differentiation and lipid metabolism (Friesen and Cowan, 2017). DCM-iPSC differentiated into fibroblasts (Ho et al., 2011) or cardiomyocytes (Siu et al., 2012; Lee et al., 2017) show increased nuclear blebbing or increased rates of apoptosis. While iPSCs from patients with skeletal muscle laminopathies are available, using them to model disease pathology has not been reported.

Numerous protocols have been developed to direct differentiation of iPSCs into myogenic cells (Loperfido et al., 2015; Kodaka et al., 2017). These approaches can be broadly categorized into either transgene-based overexpression of myogenic regulatory factors such as Pax3, Pax7 or MyoD (e.g., Darabi et al., 2012; Tedesco et al., 2012), or transgene-free systems using growth factors and small molecules to mimic myogenesis *in vitro* (reviewed in Chal and Pourquié, 2017; Kodaka et al., 2017). In this study, we differentiated three iPSC lines from patients with skeletal muscle laminopathies carrying Lamin A/C p.K32del, p.L35P, and p.R249W gene mutations into inducible myogenic cells using our published protocol (Tedesco et al., 2012; Gerli et al., 2014; Maffioletti et al., 2015). Proliferating *LMNA*-mutant inducible myogenic cells cultured as monolayers had significant abnormalities in nuclear morphology, aggregates of Lamin A/C and Lamin B1 capping. Terminal myogenic differentiation and fusion into multinucleated myotubes was not overtly affected, but mislocalization of Lamin A/C was only noted in one line, R249W. Although all *LMNA*-mutant lines demonstrated some nuclear shape defects in multinucleate myotubes grown in monolayer culture, these shape defects became significantly more pronounced when differentiated in 3D artificial muscle constructs, with an increase in the proportion of abnormally elongated nuclei.

## Results

### Differentiation of *LMNA*-mutant iPSCs into inducible myogenic cells

Three *LMNA*-mutant iPSC lines derived from patients affected by skeletal muscle laminopathies were kindly provided by Cellular Dynamics International Inc. and Cure CMD (Table 1). Episomal vector-based reprogramming was utilized to generate iPSC colonies, whose pluripotency was PCR-validated by Cellular Dynamics International Inc. with a proprietary set of genes using a Bioanalyzer. Three distinct heterozygous dominant *LMNA* mutations were represented by these iPSCs: p.K32del and p.L35P (both encoded by *LMNA* exon 1) and p.R249W (encoded by *LMNA* exon 4) (Table 1).

**Table 1:** *LMNA*-mutant iPSC mutations and patient phenotype.

***LMNA* exon**	**cDNA**	**Protein**	**Protein domain**	**Cell type**	**Affected tissue**	***LMNA*-database mutation reports^b^**	**Cell source**
1	c.94_96 delAAG	p.(K32del)	Head/ coil 1A^a^	iPS cell	Skeletal and heart muscle	5 reports: EDMD (2 cases), L-CMD (2 cases), asymptomatic (1 case)	Cure CMD and Cellular Dynamics International Inc.
1	c.104T>C	p.(L35P)	Coil 1A	iPS cell	Skeletal and heart muscle (L-CMD)	1 report: L-CMD (1 case)	Cure CMD and Cellular Dynamics International Inc.
4	c.745C>T	p.(R249W)	Coil 2, ERK 1/2 binding domain	iPS cell	Skeletal and heart muscle (LGMD1B)	9 reports: L-CMD (6 cases), EDMD (2 cases), Striated muscle laminopathy (1 case)	Cure CMD and Cellular Dynamics International Inc.

a The beginning of coil 1A is often stated as amino acid 34 (Capell and Collins, 2006; Captur et al., 2017; UMD-LMNA, 2018), however others report this as position 31 (Scharner et al., 2010; Tan et al., 2015; Zwerger et al., 2015). Thus, K32del has been reported to be in the head domain (UMD-LMNA, 2018), or in coil 1A (Bank et al., 2011). Since the crystal structure of the head domain and coil1A has not been solved, the exact domain that this mutation is in remains uncertain.

b Laminopathy disorders reported to be caused by these mutations in the LMNA-Universal mutation database http://www.umd.be/LMNA/.

*EDMD, Emery-Dreifuss muscular dystrophy; L-CMD, LMNA-related congenital muscular dystrophy*.

**Figure 1:**
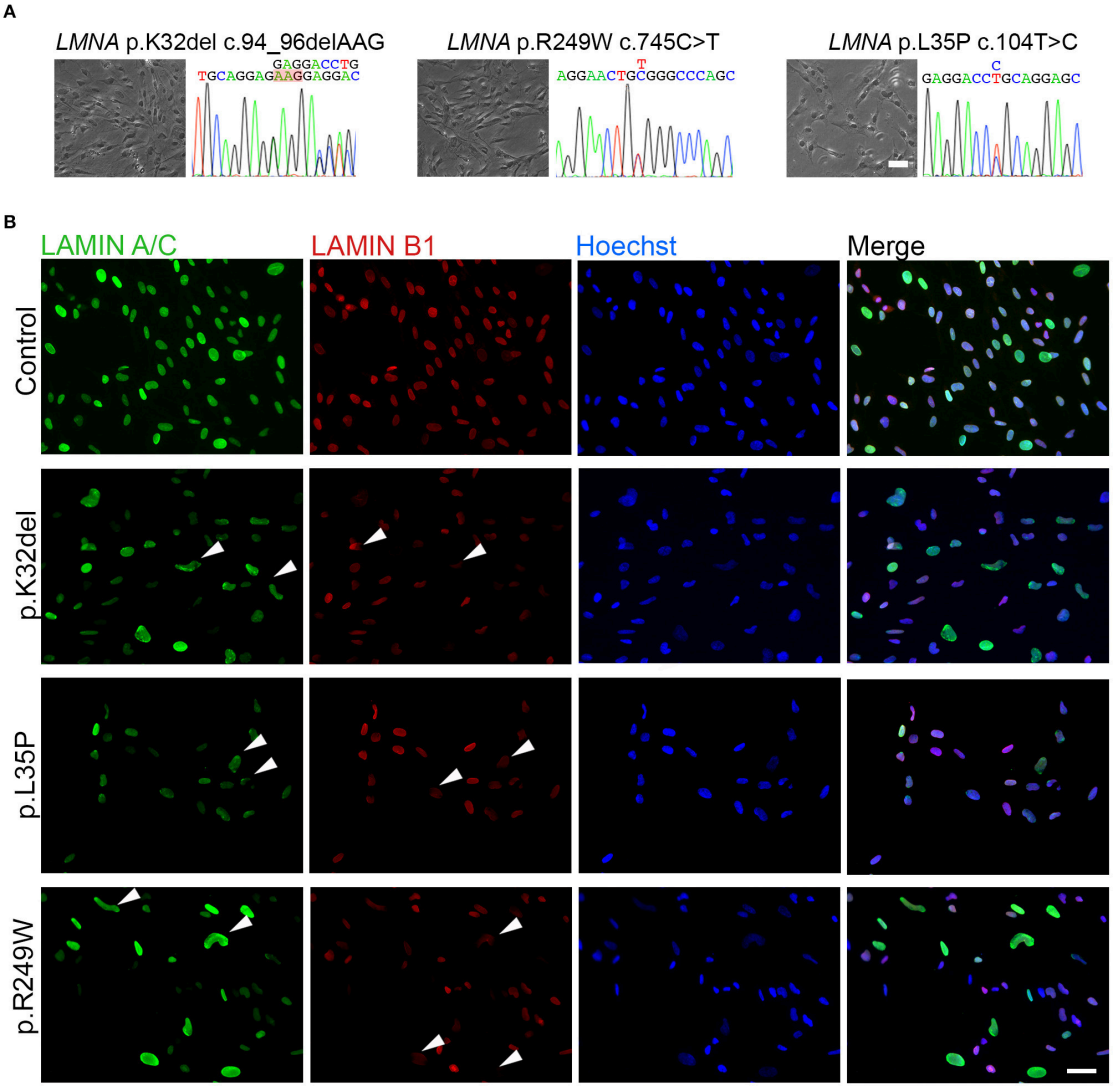
Differentiated *LMNA*-mutant human iPSCs express Lamin A/C and Lamin B1 and many have an abnormal nuclear shape. **(A)** Phase contrast images of the three *LMNA* mutant inducible myogenic cell lines K32del, R249W and L35P next to their respective electropherograms confirming the expected heterozygous, dominant, pathogenic *LMNA* mutations. **(B)** Representative immunofluorescence panel for Lamin A/C and Lamin B1 proteins showing abnormally-shaped nuclei and mislocalized Lamin B1 in all *LMNA* mutant lines (examples highlighted by arrowheads). Nuclei were counterstained with Hoechst. Scale bar: 50 μm.

*LMNA*-iPSC were differentiated into inducible myogenic cells using our protocol that generates an expandable population of mesodermal cells similar to mesoangioblasts (i.e., HIDEMs: Human iPSC Derived Mesoangioblast-like cells), which can be efficiently induced to terminal skeletal myogenic differentiation by transient expression of the myogenesis regulator MyoD (Tedesco et al., 2012; Gerli et al., 2014; Maffioletti et al., 2015). After a 3-week commitment from iPSCs to HIDEMs, *LMNA*-mutant cells morphologically resembled control HIDEMs derived from healthy donors (Maffioletti et al., 2015). Sequencing confirmed that the lines contained the expected heterozygous pathogenic *LMNA* mutations (Figure 1A). HIDEMs typically exhibit a characteristic profile of cell surface markers (Tedesco et al., 2012; Maffioletti et al., 2015), and as expected, the three *LMNA*-mutant lines expressed CD146, CD13, CD44, and CD49b at levels similar to previously generated control cells, and were negative for CD56, CD45 and CD31 (Supplementary Figure 1A). Immunofluorescence for the pluripotency-associated markers SOX2 and OCT3/4 was negative in all three *LMNA*-mutant lines demonstrating lack of residual pluripotent cells in the differentiated population (Supplementary Figure 1B). There was also an absence of Lamin A/C expression in *LMNA*-mutant iPSC colonies, with faint expression only in differentiating cells near the edges of those colonies (Supplementary Figure 1C). Derivatives of these three *LMNA*-mutant iPSC lines have also been recently used in 3D cultures (Maffioletti et al., 2018) and we provide here a detailed analysis of their use for modeling skeletal muscle laminopathies in both conventional cultures and 3D platforms.

### *LMNA*-mutant inducible myogenic cells have deformed nuclei

**Figure 2:**
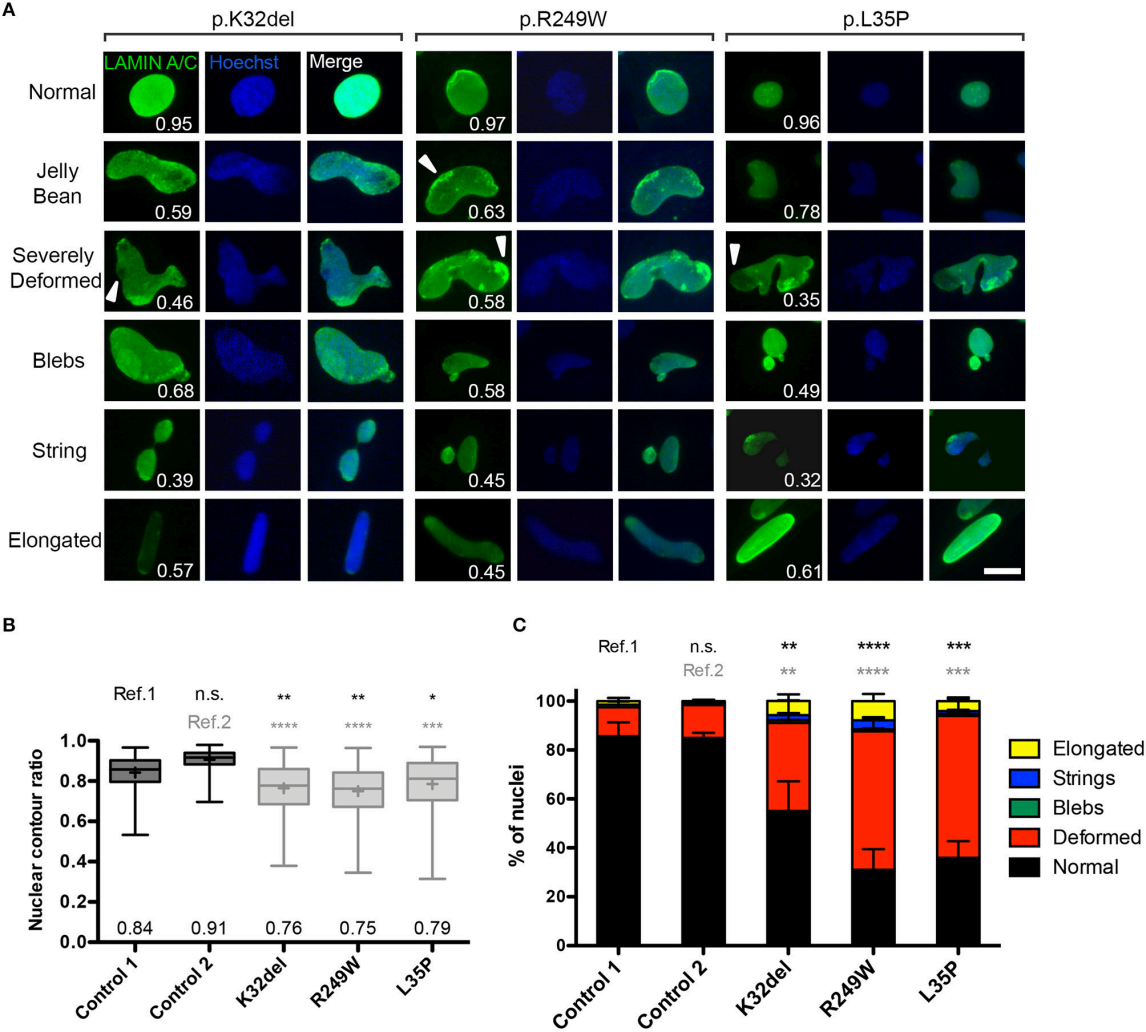
*LMNA*-mutant human iPSC-derived inducible myogenic cells have nuclei with abnormal morphology. **(A)** Representative examples of immunofluorescence showing nuclear morphology of *LMNA*-mutant HIDEMs K32del, R249W, and L35P (high magnification pictures from Figure 1B). Abnormal nuclear morphologies were classified into five categories: jelly bean, severely deformed, blebs, string or elongated, and examples of each are shown for each cell line, with their corresponding nuclear contour ratio values. Cells with normally-shaped circular/oval nuclei typically have a nuclear circularity of 1–0.79, whereas nuclear deformity reduces this ratio (indicated on each panel). Some mislocalization of Lamin A/C is also evident (white arrowheads). Scale bar: 20 μm. **(B)** Nuclear deformity in *LMNA*-mutant HIDEMs quantified using the nuclear contour ratio. One-way analysis of co-variance (ANOVA) with Tukey's *post-hoc* comparisons performed on the average values of the three repeats compared to each control line (*n* = 3, 152–334 nuclei assessed per repeat passage for each cell line); ^*^p < 0.05, ^**^p < 0.01, ^***^p < 0.001, ^****^p < 0.0001. Data are box plots generated from all circularity values across three repeats combined (a total of 663–755 nuclei were analyzed per cell line); whiskers: minimum and maximum values, +: average (specified also at the bottom of each column). **(C)** Quantification of prevalence of nuclear shape abnormalities, as well as normal shaped nuclei (circular or oval). Nuclei were considered elongated if they were >25 μm along their major axis. When present, nuclei with more than one type of abnormality were counted twice (e.g., blebs and elongated), so totals can exceed 100%. Number of normal shaped nuclei was compared between *LMNA*-mutant HIDEMs and control 1 (ref.1) and control 2 (ref.2). Between 81 and 167 nuclei were analyzed per cell line per repeat, apart from one repeat of control 1 where only 51 nuclei were assessed (a total of 248–355 nuclei were assessed per cell line). Statistics were performed on the average values of the three repeats (*n* = 3) compared to each control line, using one-way analysis of co-variance (ANOVA) with Tukey's *post-hoc* comparisons; ^*^p < 0.05, ^**^p < 0.01, ^***^p < 0.001, ^****^p < 0.0001.

Immunolabeling HIDEMs revealed that the expression of Lamin A/C and Lamin B1 became detectable as iPSCs differentiated into inducible myogenic cells. However, amongst the *LMNA*-mutant cells with normal circular/oval nuclei shaped were numerous cells with deformed nuclei (non-oval), not observed in control HIDEMs (Figure 1B). A range of morphological abnormalities was observed in *LMNA*-mutant HIDEMs, which we categorized as deformed (“jellybean” in shape or severely deformed), blebs, strings, or elongation (defined as >25 μm in length) (examples in Figure 2A). Such deformities in *LMNA*-mutant cells were measured using the nuclear contour ratio (Scharner et al., 2011, 2015), which measures how a shape deviates from a circle, with 1 representing a perfect circle and decreasing values representing an increasing level of deformity. Control cell values typically ranged from 1 to 0.79; a range describing typical circular to oval-shaped nuclei within a monolayer culture. By contrast, the mean nuclear contour ratio of all three *LMNA*-mutant HIDEM lines was significantly lower (p.K32del 0.76 ± 0.02 s.d. *p* = 0.0041 vs. control 1 and *p* < 0.0001 vs. control 2; R249W 0.75 ± 0.01 s.d. *p* = 0.0013 vs. control 1 and *p* < 0.0001 vs. control 2; L35P 0.79 ± 0.03 s.d. *p* = 0.0256 vs. control 1 and *p* = 0.0003 vs. control 2; control 1 mean 0.84 ± 0.03 s.d. and control 2 mean 0.91 ± 0.01 s.d.; control 1 vs. control 2 *p* = 0.0511. *n* = 3 one-way ANOVA, Tukey's multiple *post-hoc* comparisons) (Figure 2B).

We then quantified the proportion of each of these types of abnormality, and found that “deformed” was the most frequent shape abnormality (jelly bean and severely deformed), followed by a much smaller proportion of nuclei showing blebs, strings, and elongation. There were significantly fewer normal shaped nuclei in all *LMNA*-mutant lines compared to both control 1 and control 2 (p.K32del 55.1 ± 4.0% s.d. *p* = 0.0048 vs. control 1 and *p* = 0.0057 vs. control 2; R249W 31.0 ± 7.8% s.d. *p* < 0.0001 vs. control 1 and *p* < 0.0001 vs. control 2; L35P 36.0 ± 3.5% s.d. *p* = 0.0001 vs. control 1 and *p* = 0.0001 vs. control 2; control 1 mean 85.6% ± 1.9% s.d. and control 2 mean 85.0 ± 2.0% s.d.; control 1 vs. control 2 *p* > 0.9999. *n* = 3 one-way ANOVA, Tukey's multiple *post-hoc* comparisons; Figure 2C).

In summary, Lamin A/C and B1 are expressed upon differentiation of *LMNA*-mutant iPSCs into inducible myogenic cells, but significantly more deformed nuclei are present in cells of each of the three pathogenic genotypes analyzed.

### Nuclear envelope proteins are mislocalized in *LMNA*-mutant inducible myogenic cells

**Figure 3:**
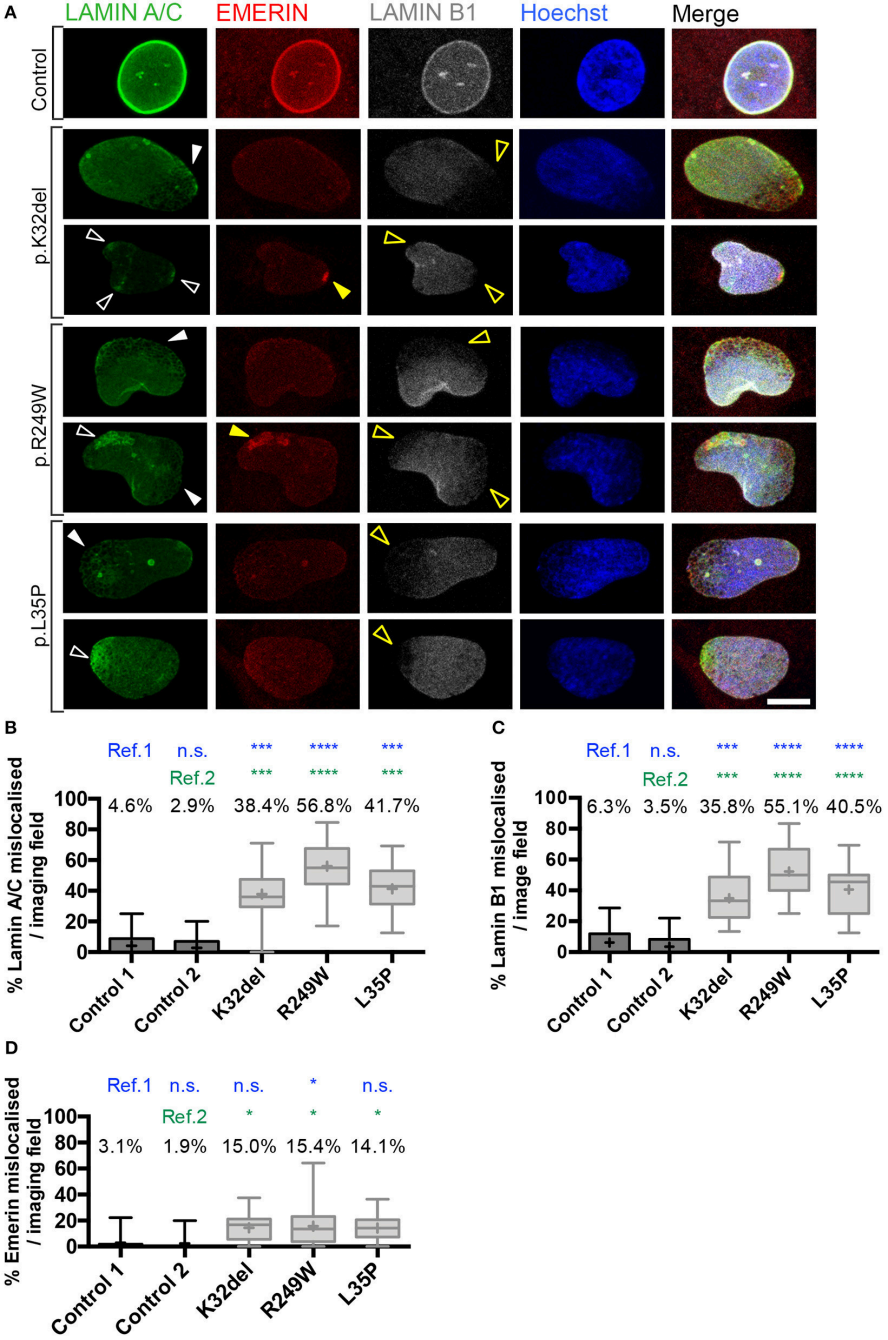
Confocal microscopy analysis in *LMNA*-mutant human iPSC-derived inducible myogenic cells shows mislocalization of Lamin A/C and Lamin B1. **(A)** Immunofluorescence for Lamin A/C, Emerin and Lamin B1 with Hoechst nuclear counterstain, showing aggregates of Lamin A/C protein in K32del, R249W, and L35P *LMNA*-mutant lines using analysis by confocal microscopy. Aggregates were either honeycomb-like in appearance (white closed arrow) or bright foci (white open arrow). Areas with Lamin A/C honeycombs/foci display a corresponding lack of Lamin B1 (yellow open arrows). Emerin-containing foci were also detected in some areas containing Lamin A/C foci (yellow closed arrow), however areas with honeycombs of Lamin A/C did not have observable corresponding honeycombs of Emerin, however Emerin immunolabeling was weak and so may be unable to detect honeycombing. Scale bar represents 10 μm. **(B)** Quantification of the proportion of *LMNA*-mutant HIDEMs with mislocalization of Lamin A/C. All *LMNA*-mutant HIDEMs had significantly more aggregates of Lamin A/C than each control cell line (ref.1 and 2) **(C)** *LMNA*-mutant HIDEMs had a significantly higher proportion of nuclei with Lamin B1 capping (absence at poles of nuclei) compared to control (ref.1 and 2). **(D)** Emerin mislocalization was significant for K32del or L35P in comparison to control 2 (ref.2) but not to control 1 (ref.1). In R249W it was significant compared to both control 1 and 2 (ref.1 and 2). We therefore judged overall this phenotype was only robust in R249W. Lamin A/C, Lamin B1 and Emerin data were statistically analyzed together using one-way analysis of co-variance (ANOVA), with Tukey's *post-hoc* comparisons. This analysis was performed on the average proportion of mislocalized from three passages per cell line (*n* = 3, between 82–178 cells analyzed per passage per cell line); ^*^p < 0.05, ^***^p < 0.001, ^****^p < 0.0001 compared to each control. Data are box plots generated from the proportion of nuclei with mislocalized Lamin A/C per imaging field (24–33 imaging fields per cell line combined from three repeats, together containing 290–429 nuclei); whiskers: minimum and maximum values, +: average of all imaging fields.

Immunofluorescence analysis indicated that Lamin A/C was also present in foci and honeycomb patterns; in addition, there were cells where Lamin B1 was missing from areas of the nucleus (capping). To better investigate mislocalization of Lamin A/C and B1, *LMNA*-mutant lines were imaged by confocal microscopy. Cells were also immunolabeled for Lamin A/C, Lamin B1, and Emerin, inner nuclear membrane protein that associates with the nuclear lamina, whose localization is dependent on Lamin A (Vaughan et al., 2001). Confocal imaging showed multiple nuclei with aggregates of Lamin A/C, which appeared as bright foci or in honeycomb patterns (Figure 3A). The proportion of Lamin A/C aggregates (honeycomb/foci) was significantly higher in each of the three differentiated *LMNA*-mutant iPSCs compared to each control cell line, with p.K32del mean 38.4 ± 9.6% s.d. (*p* = 0.0009 vs. control 1, *p* = 0.0006 vs. control 2), p.R249W mean 56.8% ± 10.4 s.d. (*p* < 0.0001 vs. control 1, *p* < 0.0001 vs. control 2), p.L35P mean 41.7 ± 3.0% s.d. (*p* = 0.0004 vs. control 1, *p* = 0.0003 vs. control 2), compared to control 1 mean 4.6% ± 4.0 s.d. and control 2 mean 2.9% ± 2.5 s.d. with no significant difference between control 1 and 2 (*p* = 0.9977) (one-way ANOVA with Tukey's multiple comparison test, *n* = 3; Figure 3B).

In nuclear regions with Lamin A/C aggregates, there was typically an absence of Lamin B1 (capping; Figure 3A). The proportion of cells with nuclear regions lacking Lamin B1 was significantly higher in *LMNA*-mutant HIDEMs compared to both control cell lines (K32del mean 35.8 ± 7.1% s.d. *p* = 0.0003 vs. control 1, *p* = 0.0001 vs. control 2; R249W mean 55.1 ± 6.4% s.d. *p* < 0.0001 vs. control 1, *p* < 0.0001 vs. control 2; L35P mean 40.5 ± 5.2% s.d. *p* < 0.0001 vs. control 1, *p* < 0.0001 vs. control 2; control 1 mean 6.3 ± 2.4% s.d.; control 2 mean 3.5 ± 3.1% s.d.; with no significant difference between controls *p* = 0.9630, one-way ANOVA with Tukey's multiple comparison test, *n* = 3; Figure 3C).

Occasional foci of Emerin in the *LMNA*-mutant lines could be seen; however, even if an increased trend could be observed in all *LMNA*-mutant lines compared to control cells, differences were only statistically significant compared to each control cell line in R249W. K32del and L35P were significant compared to control 2 only, but since they were not significant compared to control 1, Emerin mislocalization was not considered to be a robust phenotype in these lines (K32del 15.0 ± 1.2% s.d. *p* = 0.0553 vs. control 1, *p* = 0.0335 vs. control 2; R249W 15.4 ± 7.1% *p* = 0.0474 vs. control 1, *p* = 0.0288 vs. control 2; L35P 14.1 ± 4.0% s.d. *p* = 0.0795 vs. control 1, *p* = 0.0483 vs. control 2; control 1 3.1 ± 1.2% s.d. control 2 1.9 ± 1.9% s.d., differences between controls n.s. *p* = 0.9973, one-way ANOVA with Tukey's *post hoc* comparisons, *n* = 3; Figure 3D). Thus, *LMNA*-mutant HIDEMs have significant aggregates (foci, honeycombing) of Lamin A/C, as well as regions exhibiting loss (capping) of Lamin B1, typically in regions with Lamin A/C aggregates. Emerin however, only displays significant aggregates (foci) in R249W.

Confocal imaging in control 1 HIDEMs also showed a clear bright rim of peripheral nuclear Lamin A/C as well as a comparatively weaker signal of nucleoplasmic Lamin A/C. This effect was seen to a lesser extent in control 2 (Supplementary Figure 2). Distribution of Lamin A/C within the nucleoplasm was homogenous with a few brighter spots corresponding to nucleoli. In *LMNA*-mutant HIDEMs however, numerous cells lacked this brighter peripheral Lamin A/C rim, occurring in cells with and without aggregates. Differences in nucleoplasmic versus peripheral Lamin A/C was quantified using a ratio of the average fluorescence intensity of the nuclear periphery divided by the average fluorescence intensity of the nucleoplasm (Supplementary Figures 2A,B): a ratio above 1 indicates that more Lamin A/C is located at the nuclear periphery than in the nucleoplasm. Control 1 cells had a significantly higher Lamin A/C peripheral localization ratio than all *LMNA*-mutant lines indicating that Lamin A/C was not correctly localized to the nuclear lamina (Supplementary Figures 2C,D). However control 2 cells only had a significantly higher peripheral localization ratio than L35P. In addition control 1 and control 2 peripheral localization ratios were significantly different from each other indicating a degree of variability in this phenotype between healthy cell lines. We therefore considered the significance of this *in vitro* phenotype to be unresolved in HIDEMs.

We also investigated the peripheral localization ratio for Lamin B1 and Emerin. For Lamin B1, there were no consistent differences between controls and each patient-derived cell line (Supplementary Figures 3A–D). For Emerin, Control 1 generally showed significant differences compared to the mutant times, but control 2 did not (Supplementary Figures 3E–H), so we again could not resolve the significance of this phenotype in HIDEMs.

### *LMNA*-mutant R249W iPSCs exhibit nuclear abnormalities upon terminal skeletal myogenic differentiation

**Figure 4:**
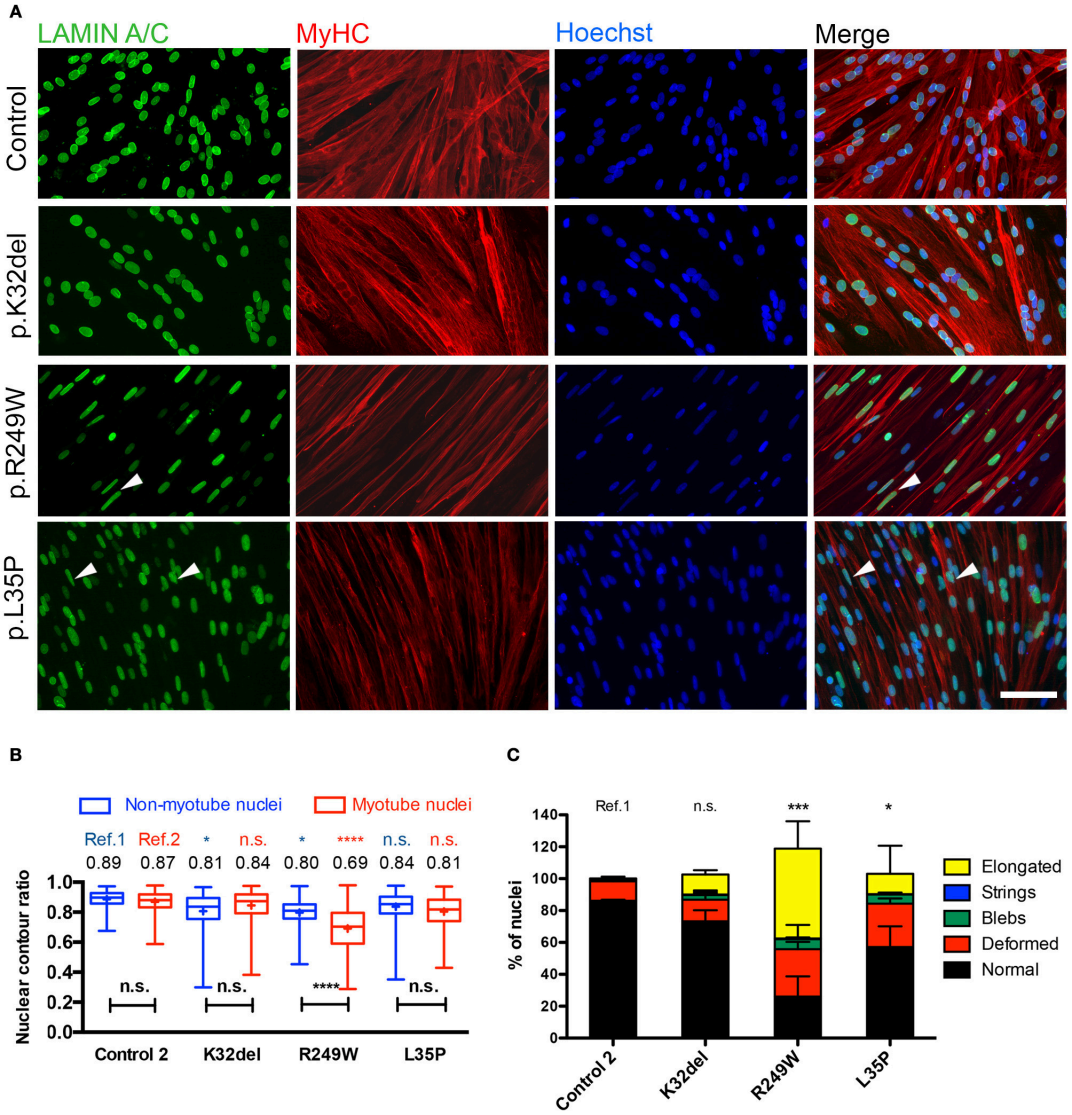
Terminal skeletal myogenic differentiation of *LMNA*-mutant iPSC-derived myotubes in monolayer culture and analysis of nuclear circularity and shape abnormalities. **(A)** Representative immunofluorescence for Lamin A/C and Myosin Heavy Chain (MyHC) on K32del, R249W, and L35P iPSCs HIDEMs induced to terminal myogenic differentiation by transient expression of a lentivirally-delivered, tamoxifen-inducible MyoD-ER transgene. Elongated nuclei and nuclear blebbing were visible (arrowheads). Scale bar: 100 μm. **(B)** Quantification of the average nuclear circularity of terminally differentiated *LMNA*-mutant iPSCs by measuring the contour ratio of nuclei within myotubes and those outside of myotubes. Refs = control. Data analyzed with two-way repeat measures ANOVA, with Tukey's *post-hoc* test for comparisons to control, and Sidak's *post-hoc* comparison for comparisons within each line. *n* = 3 independent experimental replicates, with 100–270 nuclei analyzed per cell line per repeat, except for one repeat in K32del where only 57 nuclei were found outside of myotubes. ^*^p < 0.05, ^****^p < 0.0001. Data are box plots with the individual circularity values from all three repeats combined (275–715 nuclei per cell line in total). Whiskers: min and max values; +: average of all values. **(C)** Quantification of the prevalence of myonuclear shape abnormalities based on criteria established in HIDEMs (Figure 2). When present, nuclei with more than one type of abnormality were scored twice (e.g., blebs and elongated), so totals exceed 100%. The number of normally shaped nuclei was compared between *LMNA*-mutant HIDEMs and control 2 (ref). Between 78 and 348 nuclei were analyzed per cell line per repeat (a total of 260–710 nuclei were assessed per cell line). Statistics were performed on the average values of the three repeats (*n* = 3) compared to each control line, using one-way analysis of co-variance (ANOVA) with Tukey's *post-hoc* comparisons; ^*^p < 0.05, ^***^p < 0.001.

Skeletal myogenic differentiation of control and *LMNA*-mutant HIDEMs was stimulated by tamoxifen-induced nuclear translocation of a lentivirally-delivered MyoD-ER transgene (Maffioletti et al., 2015). All *LMNA*-mutant lines and control 2 successfully differentiated with similar dynamics to form multinucleated myotubes, 4–6 days after induction. Differentiated cultures were immunolabeled for Lamin A/C and the sarcomeric protein Myosin Heavy Chain (MyHC) (Figure 4A). It was apparent that fewer nuclear shape deformities were present in the differentiated *LMNA*-mutant myotubes compared to their undifferentiated counterparts. Elongated nuclei could be seen in R249W and occasionally in L35P (Figure 4A). The nuclear contour ratio was used to quantify nuclear shape deformities in differentiated *LMNA*-mutant cultures. A nucleus was considered to be within a myotube based on three criteria: (1) within a MyHC-positive structure; (2) if that structure was multinucleated; (3) if there was nuclear exclusion of MyHC immunosignal.

Quantification demonstrated that only R249W mutant had myonuclei with a significantly lower contour ratio than control myonuclei (Figure 4B, red; K32del 0.84 ± 0.03 s.d. *p* = 0.7704, R249W 0.69 ± 0.03 s.d. *p* < 0.0001, L35P 0.81 ± s.d. *p* = 0.1382, control 0.87 ± 0.03 s.d, two–way repeat measures ANOVA with Tukey's multiple comparisons, *n* = 3). Comparisons of non-myonuclei between control and *LMNA*-HIDEMs showed that R249W and K32del, but not L35P, had significantly deformed nuclei compared to control (Figure 4B, blue; K32del 0.81 ± 0.05 s.d. *p* = 0.0470, R249W 0.80 ± 0.04 s.d. *p* = 0.0163, L35P 0.84 ± 0.01 s.d. *p* = 0.2728, control 0.89 ± 0.03 s.d, two–way repeat measures ANOVA with Tukey's multiple comparisons, *n* = 3). Furthermore, comparisons within each line showed R249W myotube nuclei were significantly deformed compared to those outside of the myotube, whereas differences within K32del, L35P and control were not significant (mean differences: K32del 0.034 *p* = 0.0596, R249W −0.1038 *p* < 0.0001, L35P −0.027 *p* = 0.1475, control −0.016 *p* = 0.5461, two–way repeat measures ANOVA with Sidak's multiple comparisons, *n* = 3).

Analysis of the proportion of normal shaped nuclei in mutant myotubes and stratification into the type of shape abnormalities showed that R249W and L35P had significantly fewer normal shaped myotube nuclei compared to control (K32del 73.3 ± 7.0% s.d. *p* = 0.4195, R249W 26.1 ± 12.7% s.d. *p* = 0.0003, L35P 57.1 ± 13.0% s.d. *p* = 0.0265, control 86.2 ± 0.7% s.d, one–way repeat measures ANOVA with Tukey's multiple comparisons, *n* = 3; Figure 4C). In R249W this was primarily due to the presence of abnormally elongated nuclei, in L35P it was mainly due to deformed, and to a lesser extent elongated, nuclei. Thus only R249W *LMNA*-mutant iPSCs produced terminally differentiated myonuclei with both an abnormal nuclear contour ratio and increased proportion of shape deformities, mainly elongation. L35P had a significant proportion of abnormal shaped nuclei in terminally differentiated myonuclei, however these shape deformities only produced a subtle difference in nuclear contour ratios. K32del did not produce any significant shape defect in terminally differentiated myonuclei.

### *LMNA*-mutant R249W iPSC-derived myotubes have mislocalized lamin A/C

**Figure 5:**
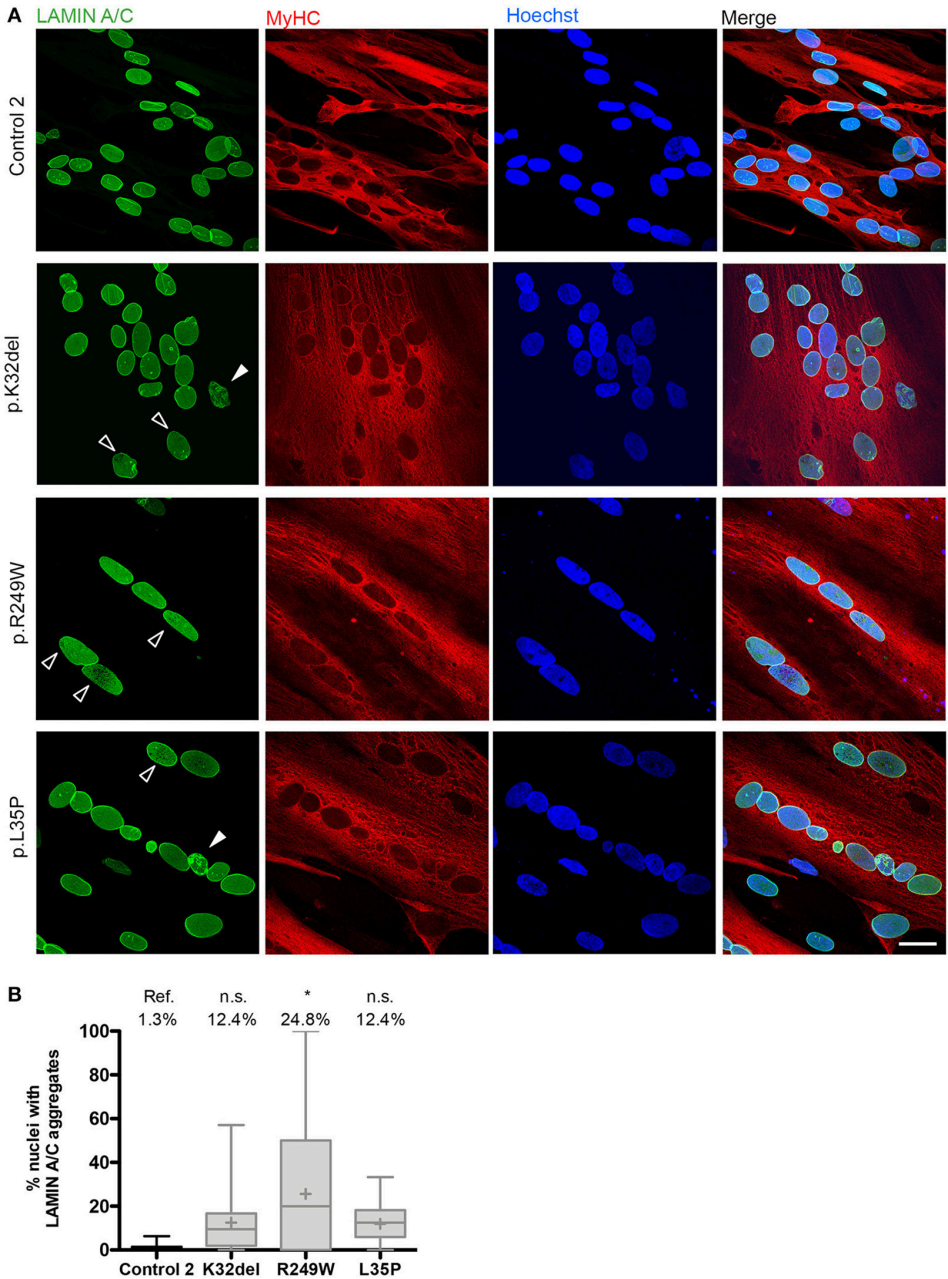
*LMNA*-R249W produces Lamin A/C aggregates upon terminal skeletal myogenic differentiation of *LMNA*-mutant iPSCs in monolayer cultures. **(A)** Immunofluorescence for Lamin A/C and MyHC, with a Hoechst nuclear counterstain, showing aggregates of Lamin A/C protein in K32del, R249W, and L35P *LMNA*-mutant myotubes upon confocal microscopy analysis. Aggregates were either honeycomb-like in appearance (white open arrow) or bright foci (white closed arrow). **(B)** Although aggregates of Lamin A/C were seen in all *LMNA*-mutant myonuclei, only R249W was significant compared to control (ref.). One-way analysis of co-variance (ANOVA) with Tukey's *post-hoc* comparisons, on the average of three passages (*n* = 3, between 74 and 156 myonuclei analyzed per passage per cell line); ^*^p < 0.05 compared to control myotubes. Data are plotted as box plots generated from the proportion of nuclei with mislocalized Lamin A/C per imaging field (21–56 imaging fields per cell line combined from three repeats, together containing 381 myonuclei); whiskers: minimum and maximum values, +: average of all imaging fields. Scale bar: 30 μm.

We next investigated if the Lamin A/C mislocalization seen in *LMNA*-mutant HIDEMs remained in myotubes. Lamin A/C honeycombing and foci were present in all *LMNA*-mutant lines (Figure 5A). However, the proportion of nuclei exhibiting these mislocalizations were lower than in *LMNA*-mutant undifferentiated HIDEMs, and only statistically significant in R249W compared to control (K32del 12.4 ± 1.2% s.d. *p* = 0.3254, R249W 24.8 ± 14.4% s.d. *p* = 0.0201, L35P 12.4 ± 1.9% s.d. *p* = 0.3123, control 1.3 ± 0.2% s.d. *n* = 3, one-way ANOVA with Tukey's *post-hoc* comparisons; Figure 5B).

Lamin A/C can relocate to the nuclear periphery during myoblast to myotube terminal differentiation, and mutations in Lamin A/C can lead to a failure of this process (Markiewicz et al., 2005). We assessed whether the *LMNA*-mutant lines were capable of this relocalization. As in *LMNA*-mutant HIDEMs differences between *LMNA*-mutant myonuclei and control were not significant (Supplementary Figures 2E,F). In addition, comparisons between HIDEMs and differentiated myotubes showed that all *LMNA*-mutant cells had significantly more Lamin A/C located at the nuclear lamina in myonuclei compared to HIDEMs (mean difference between myonuclei minus HIDEMs within each line: K32del 0.18 *p* = 0.0427, R249W 0.18 *p* = 0.0475, L35P 0.3 *p* = 0.0009, control 0.15 *p* = 0.1175, *n* = 3 two-way ANOVA with Sidak's *post-hoc* comparison) (Supplementary Figures 2G,H). Together, these results demonstrate that during terminal myogenic differentiation the *LMNA*-mutant cell lines assessed do not completely fail to relocate and assemble Lamin A/C into the nuclear lamina. In addition amongst the genotypes studied, only R249W had significant Lamin A/C mislocalization (honeycomb/foci).

### Three-dimensional culture enhances *in vitro* modeling of disease-associated nuclear abnormalities upon terminal myogenic differentiation of *LMNA*-mutant iPSCs

Standard skeletal myogenic differentiation in monolayer of *LMNA*-mutant iPSCs demonstrates consistent disease-associated phenotypes in only one of the three pathogenic genotypes. Therefore, we investigated whether sensitivity of our platform could be enhanced by altering culture conditions, such as differentiating cells in three-dimensions (3D), to produce conditions more closely resembling the *in vivo* environment. Our recent work has shown that these *LMNA*-mutant iPSCs exhibit nuclear shape abnormalities when terminally differentiated in 3D artificial muscle constructs (Maffioletti et al., 2018). However, the precise resolution of this 3D platform in detecting disease-associated phenotypes in comparison with monolayers, its mutation-specific sensitivity and ability to distinguish location of abnormal nuclei (i.e., myotube from non-myotube nuclei) are unknown.

**Figure 6:**
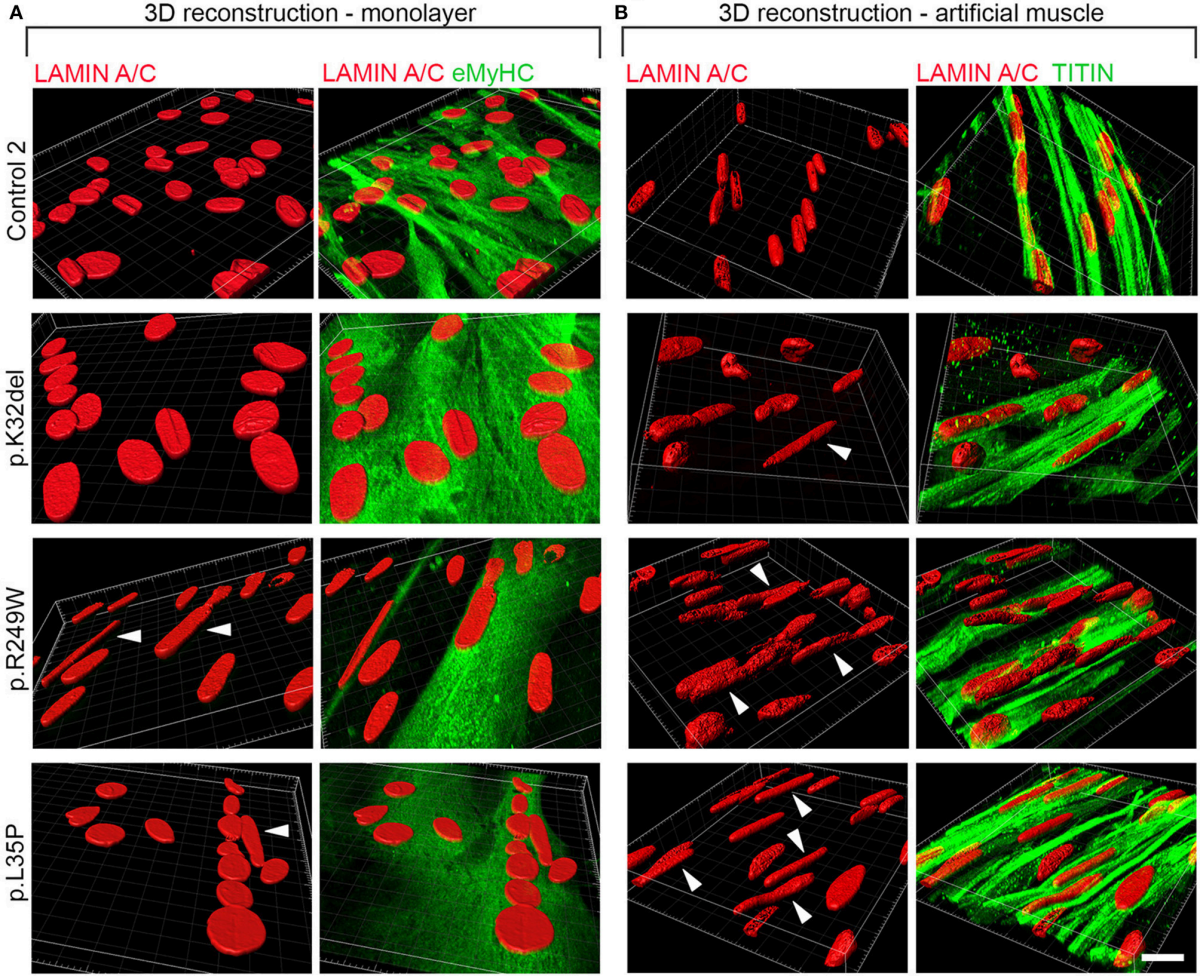
Abnormally elongated nuclei characterize *LMNA*-mutant iPSC-derived myotubes differentiated in either monolayer culture or as 3D artificial muscles. *LMNA*-mutant HIDEMs K32del, R249W, and L35P were either terminally differentiated into multinucleated myotubes in **(A)** monolayer cultures or **(B)** within 3D artificial muscles. Three dimensional computer-generated reconstructions from confocally-imaged cells immunolabeled for Lamin A/C and the myogenic markers embryonic myosin (eMyHC) or Titin. Arrows head: abnormally elongated nuclei. Scale bar: 20 μM.

**Figure 7:**
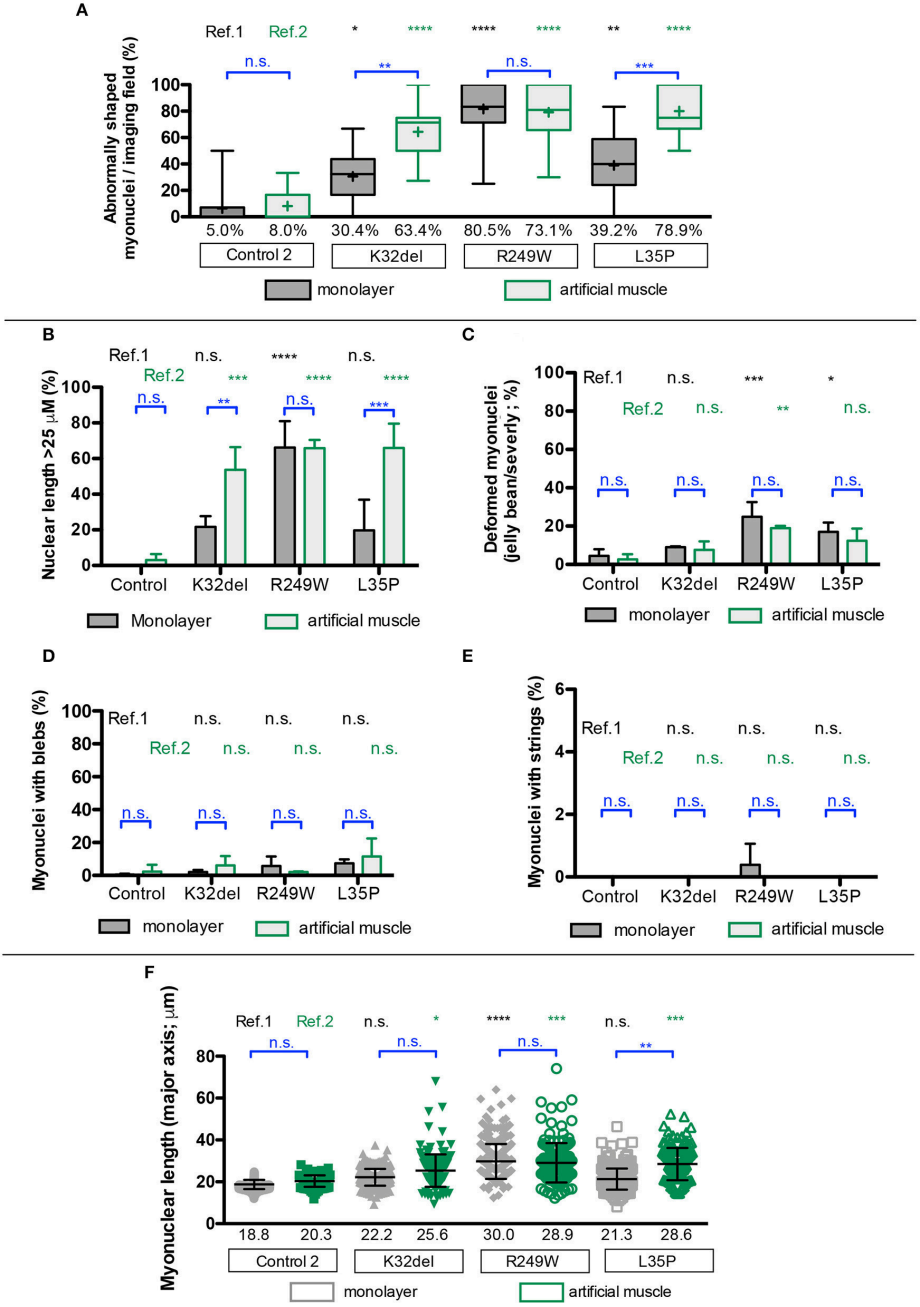
Disease-associated nuclear abnormalities are more prevalent upon terminal myogenic differentiation of *LMNA*-mutant iPSCs in three-dimensional cultures. **(A)** Abnormal K32del, R249W, and L35P myotube nuclei were assessed from the three dimensional computer-generated reconstructions shown in Figure 6, based on shape abnormality criteria established in 2D image analysis in *LMNA*-mutant HIDEMs (Figure 2). Three statistical comparisons of the myonuclei are shown: *LMNA*-mutant to control within monolayer culture (ref.1, black), within artificial muscles *LMNA*-mutant to control (ref.2, green), within each line comparisons between 2D and 3D artificial muscle (blue). Data analyzed together using two-way ANOVA with Tukey's *post-hoc* comparisons for comparisons to control, and Sidak's for comparisons within lines on the average of three passages (*n* = 3, for monolayer cultures 82–154 myonuclei were analyzed per passage per cell line with a total of 272–410 per cell line, for artificial muscle 37–70 nuclei were analyzed, totaling 124–171 per cell line). Data are shown as box plots of proportion of abnormal nuclei per imaging field from all three repeats (22–44 total imaging fields per cell line combined from three repeats); whiskers: minimum and maximum values, +: average of all imaging fields. Values shown on graph: average of three repeats used for statistical analysis. **(B–E)** Quantification of the different types of shape abnormality shown in **(A)**, based on criteria established in *LMNA*-mutant HIDEMs (Figure 2). As in **(A)**, three types of comparisons are shown. Nuclei with more than one type of shape abnormality (e.g., blebs and elongation) were counted twice, therefore totals can exceed 100%. Data analyzed together using two-way ANOVA with Tukey's *post-hoc* test for comparisons to control, and Sidak's *post-hoc* test for comparisons within lines (*n* = 3). **(F)** Myonuclear length along the major nuclear axis. As in **(A)**, three types of comparisons were performed. Data analyzed using two-way ANOVA with Tukey's *post-hoc* test for comparisons to control, and Sidak's *post-hoc* test for comparisons within lines (*n* = 3). Data are shown as a scatter plot comprised of all values from three repeats combined. Bars: mean and standard deviation. Nuclei were assessed to be part of a myotube based on location within a multinucleated myosin/titin-positives structure, and nuclear exclusion of myosin/titin. Data for non-myotube nuclei within the culture are not shown. ^*^p < 0.05, ^**^p < 0.01, ^***^p < 0.001, ^****^p < 0.0001.

All *LMNA*-mutant lines differentiated in 3D artificial muscles successfully formed multinucleated myotubes expressing markers of terminal skeletal myogenic differentiation such as embryonic MyHC or titin (Supplementary Figure 4). Three-dimensional computer-generated reconstruction of confocally-imaged cell monolayers (Figure 6A) or 3D artificial muscle constructs (Figure 6B) showed numerous elongated myonuclei within the 3D artificial muscle constructs in all *LMNA*-mutant lines (Figure 6 and Supplementary Videos). Quantification of abnormal shaped nuclei on 3D reconstructed images using the criteria established with 2D imaging of *LMNA*-mutant HIDEMs and monolayer myotubes (Figures 2C, 4C) showed that both monolayer and 3D artificial muscle had significantly more abnormal nuclei compared to their respective controls (monolayer comparisons to control: K32del 30.4 ± 4.5% s.d. *p* = 0.0263, R249W 80.5 ± 13.6% s.d. *p* < 0.0001, L35P 39.2 ±17.7% s.d. *p* = 0.0028, control 5.0 ± 3.7% s.d.; 3D artificial muscle comparisons to control: K32del 63.4 ± 10.5% s.d. *p* < 0.0001, R249W 73.1 ± 8.9% s.d. *p* < 0.0001, L35P 78.9 ± 5.1% s.d. *p* < 0.0001, control 8.0 ± 3.4% s.d.; *n* = 3 two way ANOVA with Tukey's *post-hoc* comparisons; Figure 7A). However, these differences were more pronounced in the 3D artificial muscle, with over double the number of abnormal nuclei detected in K32del and L35P cultures (Figure 7A). Comparisons within each line between the monolayer and 3D artificial muscle showed that this difference was statistically significant in K32del and L35P (Mean difference between monolayer and 3D artificial muscle within each line: K32del 33.0% *p* = 0.0031, R249W −7.4% *p* = 0.8382, L35P 39.7% *p* = 0.0005, control 3.0% *p* = 0.9929, *n* = 3 two-way ANOVA with Sidak's *post-hoc* comparison; Figure 7A). Importantly, these are the two mutations that did not produce a significant difference compared to control when assessed for nuclear contour ratio in monolayer myotubes (Figure 4B). For the R249W mutation that did produce an abnormal nuclear contour ratio in the monolayer, there was no increase of abnormal myonuclei in artificial muscles.

Stratification into types of abnormalities, showed that elongated nuclei were the most frequent type of shape abnormality observed (Figures 7B–E). This is consistent with our previous data in artificial muscles (Maffioletti et al., 2018) and in contrast to undifferentiated HIDEM cultures, where deformed (jellybean and severely deformed) were the most frequently observed morphological defect. Comparisons within each line showed that 3D cultures produced a significant increase in elongated myonuclei compared to monolayer culture for K32del and L35P (Mean difference between monolayer and 3D artificial muscle within each line: K32del 32.0% *p* = 0.0092, R249W 0.4% *p* > 0.9999, L35P 46.2% *p* = 0.0003, control 3.0% *p* = 0.9953, *n* = 3 two-way ANOVA with Sidak's *post-hoc* comparison) (Figure 7B). There was no significant increase in the other types of shape defects between monolayer and 3D artificial muscle constructs (Figures 7C–E). This demonstrates that culture of *LMNA* mutant lines as artificial muscle constructs produces a higher number of abnormal myonuclei due to an increase in the number of myonuclei demonstrating an abnormal elongation (>25 μm).

Having identified nuclear elongation as an important morphological defect, we then compared the mean length of all myonuclei assayed (Figure 7F). Average nuclear length was only significantly increased in monolayer cultures for R249W compared to control (comparisons to control: K32del 22.2 ± 1.1 μm s.d. *p* = 0.2202, R249W 30.0 ± 3.6 μm s.d. *p* < 0.0001, L35P 21.3 ± 2.4 μm s.d. *p* = 0.4621, control 18.8 ± 0.9 μm s.d.; *n* = 3 two way ANOVA with Tukey's *post-hoc* comparisons) (Figure 7F). However, in 3D artificial muscle constructs, the length of myotube nuclei was significantly higher for each of the *LMNA*-mutant lines compared to control (comparisons to control: K32del 25.6 ± 1.9 μm s.d. *p* = 0.0305, R249W 28.9 ± 2.6 μm s.d. *p* = 0.0006, L35P 28.6 ± 1.8 μm s.d. *p* = 0.0009, control 20.3 ± 0.6 μm s.d.; *n* = 3 two way ANOVA with Tukey's *post-hoc* comparisons) (Figure 7F). This corresponds to a significant increase in myonuclear length of L35P in 3D artificial muscles compared to monolayer cultures (mean difference between monolayer and artificial muscle: K32del 3.4 μm *p* = 0.2287, R249W −1.1 μm *p* = 0.9521, L35P 7.3 μm *p* = 0.0022, control 1.54 μm *p* = 0.8496, *n* = 3 two-way ANOVA with Sidak's *post-hoc* comparison).

In summary, myogenic differentiation of *LMNA*-mutant iPSCs in a 3D microenvironment, coupled with high-resolution 3D nuclear imaging, improves detection of disease-associated phenotypes in comparison to conventional monolayer cultures and unravels cellular hallmarks *in vitro* across multiple pathogenic genotypes, providing a high-fidelity modeling platform for skeletal muscle laminopathies.

## Discussion

In this study, three *LMNA*-mutant iPSC lines K32del, R249W, and L35P from patients affected by skeletal muscle laminopathies have successfully been differentiated into inducible skeletal myogenic cells and then into terminally differentiated myotubes *in vitro*. All three *LMNA*-mutant iPSC lines showed significant defects in nuclear morphology when analyzed as proliferating inducible myogenic cells. Previous studies have shown that *LMNA* mutations causing skeletal muscle laminopathies lead to defects in nuclear shape in primary patient fibroblasts (Favreau et al., 2003; Muchir et al., 2003, 2004; Tan et al., 2015), murine C2C12 myoblasts (Favreau et al., 2003; Scharner et al., 2011; Barateau et al., 2017), as well as primary human myoblasts (Bertrand et al., 2014).

The R249W mutation is known to cause abnormal nuclear shape in undifferentiated cells upon transduction into murine C2C12 and primary human myoblasts in 3D cultures (Scharner et al., 2011; Bertrand et al., 2014). Primary undifferentiated human myoblasts in 3D culture with K32del also have an elongated nuclear shape (Bertrand et al., 2014), but primary fibroblasts did not have nuclear shape abnormalities (Muchir et al., 2004). Here, we found that myotube nuclei carrying R249W had morphological defects in nuclear contour ratio when grown in monolayer culture, while L35P and K32del did not. However, all mutations produced a higher proportion of abnormal myotube nuclei compared to control monolayer cultures when assessed using three dimensional computer-generated image reconstruction, with R249W showing the highest. While abnormal cells were predominantly caused by elongated nuclei in myotubes, in proliferating inducible myogenic cells, nuclei were mainly jellybean/severely deformed.

We recently showed nuclear shape defects in K32del, R249W, and L35P myotubes in 3D artificial muscle constructs (Maffioletti et al., 2018). Here, we extended the analysis and performed mutation-specific assessment of myonuclear shape and length; moreover, we directly investigated if 3D artificial muscles provide better *in vitro* modeling of nuclear abnormalities than traditional monolayer cultures in specific LMNA genotypes. Differentiating HIDEMs in the 3D culture significantly improved modeling of the nuclear shape defects for K32del and L35P mutations, with a significant increase in the proportion of abnormally shaped myotube nuclei in the 3D artificial muscle compared to monolayer cultures. This was due to an increase in the proportion of abnormally elongated nuclei, consistent with our previous report (Maffioletti et al., 2018).

Skeletal muscle biopsies from four patients with EDMD/LGMD1B revealed that 17% of myonuclei had an abnormal nuclear shape, although there was no shape abnormality in satellite cells (Park et al., 2009). *In vivo*, elongated cardiomyocyte nuclei have been reported in heart biopsies of *Lmna*^+/−^, *Lmna*^−/−^ (Nikolova et al., 2004) as well as *Lmna*^K32del/+^ mutant mice (Cattin et al., 2013, 2016). Therefore, for the types of shape defects observed in patient muscle biopsies to become more pronounced in skeletal myotubes *in vitro*, cells may require some degree of external stress (e.g., mechanical tension found in a 3D artificial muscle environment) that mimics *in vivo* conditions. This mechanical tension could stretch the nuclei into the elongated shape, with *LMNA*-mutant nuclei less able to resist such deformation. In keeping with this notion, an external stress of electrical stimulation to induce contraction also produces a phenotype in DCM-iPSC derived cardiomyocytes which showed no overt shape abnormality at rest (Siu et al., 2012).

Why the skeletal myonuclei require a 3D environment to show a phenotype, when the proliferative myogenic cells we assessed did not, is unclear. It could be that skeletal myotubes are more resistant to deformation and so require more stress to induce a phenotype. This could potentially be due to differences in Lamin A/C expression levels or its localization to the periphery. An alternative, less likely, explanation could be that the most abnormal cells are lost during prolonged culture or might be resistant to fusing into myotubes.

K32del, R249W, and L35P mutations also caused significant honeycomb/foci aggregates of Lamin A/C in proliferating myogenic cells, consistent with previous reports showing that R249W causes disorganization of the nuclear lamina lattice resulting in loss of Lamin A from cell poles (capping) (Scharner et al., 2011). Primary fibroblasts from patients harboring the K32del mutation have honeycomb mislocalizations of Lamin A/C (Muchir et al., 2004), and we also found HIDEMs with similar mislocalization of Lamin A/C for this mutation. However, we saw that when terminally differentiated into skeletal myotubes, only R249W had nuclei with significant honeycomb/foci of Lamin A/C.

The honeycomb/foci aggregates of Lamin A/C could be due to a protein assembly defect. In support of this, *LMNA* mutations underlying DCM and EDMD cause assembly defects *in vitro* (Wiesel et al., 2008; Bank et al., 2012; Bhattacharjee et al., 2013). Furthermore, when expressed in *C. elegans*, K46del (corresponding to K32del in humans) interferes with the lateral assembly of dimeric head-to-tail polymers into antiparallel tetrameric filaments, which *in vivo* result in peripheral nuclear aggregates in post-larval tissues (Bank et al., 2011).

It is unclear why myonuclei have lower levels of Lamin A/C mislocalization than proliferative cells. It could be that nuclei with the most mislocalization are unable to fuse into myotubes or are progressively lost during culture. Alternatively, mislocalizations in proliferative nuclei could be easier to resolve due to differences in Lamin A/C expression level compared to terminally differentiated myonuclei.

All *LMNA-*mutant HIDEM lines also had low levels of Lamin A/C at the nuclear lamina, however we found this phenotype was inconsistent, as there was significant variability even between control cell lines. Only L35P consistently showed significantly less Lamin A/C at the nuclear lamina compared to both control cell lines. Nonetheless, there was no significant difference in myonuclear peripheral localization ratio between any *LMNA*-mutant line and control cells upon terminal skeletal myogenic differentiation. Furthermore, we observed relocation of Lamin A/C to the nuclear periphery in terminally differentiated skeletal myonuclei at variance with reports showing that skeletal muscle laminopathy mutations produce a defect in Lamin A/C relocation to the nuclear periphery during terminal myogenic differentiation (Markiewicz et al., 2005). Mutations in *LMNA* lead to a reduction in the amount of peripheral Lamin A/C in muscle from *Lmna*^k32del/k32del^ and *Lmna*^K32del/+^ mice (Bertrand et al., 2012; Pilat et al., 2013) or when overexpressed in murine C2C12 myoblasts (Ostlund et al., 2001; Markiewicz et al., 2005; Barateau et al., 2017). Primary human myoblasts with K32del and R249W also have Lamin A/C mislocalized to the nucleoplasm (Bertrand et al., 2014). From our results it seems that the *LMNA*-mutant lines assessed do not have a complete failure to relocate and assemble Lamin A/C to the nuclear lamina during terminal myogenic differentiation. However, the relative contribution of the WT vs. mutant *LMNA* allele to this peripheral Lamin A/C is uncertain and more sensitive quantitative methods could detect a more subtle difference between patient and control cells. We believe that due to the variability seen between controls, modeling this phenotype would benefit from the use of isogenic controls, for example generated using gene editing technologies.

Within nucleoplasm, Lamin A/C controls Rb phosphorylation, which is important in regulation of cell cycle progression and cell cycle exit during terminal myogenic differentiation. Lamin A/C deficient cells or those harboring EDMD/L-CMD mutations have defects in muscle differentiation (Favreau et al., 2004; Markiewicz et al., 2005; Frock et al., 2006; Gnocchi et al., 2011; Pilat et al., 2013). These differentiation defects are associated with a lack of proper cell cycle exit and Rb remaining hyper-phosphorylated (Favreau et al., 2004), as well as a failure of Lamin A/C to relocate from the nucleoplasm to the nuclear lamina (Markiewicz et al., 2005). However, we did not observe overt issues with differentiation into multinucleated myosin heavy chain positive myotubes. This included K32del, which causes delayed and insufficient differentiation in mouse, with a failure to upregulate myosin heavy chain during *Lmna*^K32del/K32del^ myoblast differentiation (Pilat et al., 2013). We used MyoD overexpression to drive differentiation, which could mask possible defects in the native/spontaneous skeletal myogenic differentiation programme. Indeed, MyoD can rescue impaired differentiation seen in mouse *Lmna*^−/^^−^ myoblasts (Frock et al., 2006). Recently, transgene free approaches have been devised that do not rely on over expression of MyoD (reviewed in Chal and Pourquié, 2017). Such systems might be better suited to studying disease-associated alterations in differentiation potential, and future work in our laboratories will seek to exploit this methodology.

We also observed loss of Lamin B1 in regions with aggregates of Lamin A/C, as we previously reported for R249W, which causes disorganization of the nuclear lamina lattice resulting in loss of both Lamin A and B1 from cell poles (capping) when exogenously expressed in murine C2C12 myoblasts (Scharner et al., 2011). However, we did not see any significant mislocalization of Lamin B1 from the nuclear periphery when Lamin A/C was absent, as consistent with previous reports in *Lmna*^K32del/K32del^ embryonic muscle sections where Lamin B1 is at the nuclear periphery (Bertrand et al., 2012). Abnormally shaped nuclei, together with the mislocalization of Lamin B1, could render the nuclei more susceptible to nuclear rupture. Areas without Lamin B1 are also often the sites of nuclear envelope blebbing leading to rupture, which results in loss of compartmentalization between the nucleus and the cytoplasm (Lammerding et al., 2004). Indeed, in laminopathy patient fibroblasts, abnormal shaped nuclei are associated with increased nuclear rupture (De Vos et al., 2011).

Lamin A/C mutations can cause loss of Emerin at the nuclear envelope *in vitro* when overexpressed in C2C12 myoblasts (Ostlund et al., 2001), and *in vivo* in *C. elegans* expressing K46del (equivalent to human K32del) (Bank et al., 2011). In L35P we also found significant mislocalization of Emerin from the nuclear periphery compared to one control only. However, as with Lamin A/C there was again a significant amount of variability between control cell lines and so we could not resolve this question.

In conclusion, our work shows that skeletal muscle laminopathies can be modeled using human iPSCs at different stages of differentiation. Although nuclear abnormalities could be observed in proliferating iPSC derivatives, conventional skeletal myogenic differentiation resulted in less-pronounced disease-associated phenotypes. Conversely, three dimensional nuclear analysis of 3D artificial muscles derived from *LMNA*-mutant iPSCs resulted in high-fidelity mutation-specific modeling which was superior to conventional monolayer cultures. Using *LMNA*-mutant iPSCs to model muscle laminopathies could provide the foundations for next-generation therapy screening platforms to tackle this group of severe and incurable disorders.

## Materials and methods

### Cell lines, culture, and differentiation

*LMNA*-mutant human iPSCs were kindly provided by Cellular Dynamics International Inc. (CDI; https://cellulardynamics.com) and Cure Congenital Muscular Dystrophy (CureCMD; https://www.curecmd.org). iPSCs were generated by CDI by reprogramming samples provided by CureCMD (holder of clinical information) using episomal vectors. Pluripotency of iPSC lines was validated by CDI using a proprietary set of genes using a Bioanalyzer. Culture of *LMNA*-mutant and control human iPSCs, their differentiation into inducible myogenic cells (HIDEMs), and terminal differentiation into skeletal myotubes was performed as previously described (Maffioletti et al., 2015). Three-dimensional artificial skeletal muscles were generated as recently reported (Maffioletti et al., 2018; Prüller et al., 2018), from HIDEMs differentiated within hydrogels for 7 days after tamoxifen-induced MyoD nuclear translocation.

### Immunofluorescence staining and microscopy

Cells were washed with PBS, fixed with 4% (w/v) paraformaldehyde (PFA) for 7 min, followed by a further PBS wash. Fixed cells were permeabilized for 1 h with permeabilization solution [1% bovine serum albumin (BSA) + 0.2% Triton in PBS]. Cells were then blocked for 30 min with 10% donkey or goat serum diluted in permeabilizing solution, followed by overnight incubation with primary antibodies diluted in permeabilizing solution. Unbound primary antibody was removed using three washes of 0.2% Triton in PBS. After this, cells were incubated for 1 h with secondary antibodies and Hoechst 33342 diluted in 0.2% Triton in PBS. Unbound secondary antibody was washed away with two washes of 0.2% Triton in PBS, followed by one rinse of PBS. Cells were imaged with an inverted fluorescence microscope. As a control for non-specific secondary antibody binding, a separate well was incubated with only secondary antibody.

The following primary antibodies were used: rabbit anti-SOX2 1:100 (Abcam AB97959) mouse anti-OCT3/4 1:100 (Santa Cruz sc5279), rabbit anti-NANOG 1:100 (Abcam ab80892), mouse anti-Lamin A/C 1:250 (Novocastra NCL-LAM-A/C, IgG2b) or goat anti-Lamin A/C 1:250 (for myogenic differentiation nuclear contour ratio only, Santa Cruz sc-6215), rabbit anti-Lamin B1 1:2,000 (Abcam ab16048), goat anti-Emerin 1:100 (Santa Cruz SC8086), mouse anti-myosin heavy chain 1:5 (MF20 clone which recognizes all myosin heavy chain forms, DSHB hybridoma bank), mouse anti-embryonic myosin 1:5 (F1.652 DSHB). Secondary antibodies were all AlexaFluor® (Life Technologies; 1:500); when mouse anti-Lamin A/C (Novocastra NCL-LAM-A/C, IgG2b) and mouse anti-embryonic myosin (DSHB F1.652, IgG1) were used together, IgG-specific AlexaFluor® secondary antibodies were also used.

3D hydrogels were immunolabeled as described previously (Maffioletti et al., 2018). Following fixation with 4% (w/v) PFA for 3 h at 4°C and incubation with blocking solution, the primary antibody mixture consisting of mouse anti-Titin 1:50 (DSHB 9D10, IgM) and mouse anti-Lamin A/C 1:125 (Novocastra NCL-LAM-A/C, IgG2b) was added. Next day, gels were washed with TBS six times over the course of a day and incubated with Hoechst (1:125), goat anti-mouse IgM (488) and goat anti-mouse IgG2B (546) (1:250). The day after, gels were washed again six times with TBS, mounted on an indented glass slide (Carl Roth, H884.1) in Fluoromont G mounting medium (Dako, S3023A) and imaged using a confocal microscope.

For standard fluorescence microscopy, a Leica DM16000B inverted microscope with Leica application suite advanced fluorescence software was used. For confocal fluorescence microscopy imaging the upright Leica SPE2 was used running LAS-X software, with a 63X objective and a slice size of 1 μm. Scanning parameters 1,024 × 1,024, speed of 600, zoom factor 1.5 and frame average 3. For confocal imaging, cells were plated on Labtex II chamber glass slide (Nunc). For confocal analysis in myotubes, proliferating myogenic cells were differentiated for 4–7 days on Fluodishes (35 mm, World Precision Instruments), coated with matrigel as previously described (Maffioletti et al., 2015).

### Analysis of nuclear abnormalities

To perform nuclear contour ratio analysis, abnormalities in nuclear shape on lamin A/C immunolabelled images were manually drawn around using the polygon tool in Fiji imaging software. The area and circumference measurements were used to quantify the nuclear contour ratio (4π area/circumference^2^) (Goldman et al., 2004). Cells were imaged using a 32X lens on an inverted microscope for all nuclear contour ratio quantification. Imaging fields were randomly selected, and multiple imaging fields were taken for each repeat (between 3 and 14), with between 150 and 300 cells analyzed. Three repeats of independent cell passages fixed on different days were analyzed. For each repeat the nuclear contour ratio was averaged to the number of cells analyzed.

For shape classification, myonuclei were scored as deformed (e.g., hook or jelly bean structures), elongated (major axis >25 μm), with blebs (Lamin A/C protrusions), strings (represented by thin protrusions of Lamin A/C within the nuclear structure) or normal (circular/oval with no shape defects) by a blinded researcher. A cut-off of 25 μm for abnormal nuclear elongation was chosen based on a preliminary assessment of qualitatively elongated nuclei.

For peripheral localization analysis, Lamin A/C, B1 and Emerin immunolabelled cells were imaged by confocal microscopy. A 3.5 μm stack was taken in the middle of the nuclei in the Z plane (6 slices of 1 μm thickness with a 0.7 μm overlap), and a maximum intensity projection generated. Two regions were manually drawn around using the polygon tool in Fiji imaging software: entire nuclei, and the nucleoplasmic component. Values from these regions were then used to calculate the peripheral localization ratio (mean average fluorescence of the nucleoplasm/mean average fluorescence of the nuclear periphery). Three repeats of independent cell passages were analyzed. Approximately 16 fields were taken for each repeat, with 100 cells analyzed for each. For each repeat the peripheral localization ratio was averaged to the number of cells analyzed.

Mislocalization analysis of Lamin B1 capping, and aggregates of Lamin A/C and Emerin was conducted on the same maximum projections used in the Lamin A/C peripheral localization analysis. Aggregates of Lamin A/C were scored in two categories: honeycomb and foci. Honeycomb contained multiple patches where lamin A/C staining was absent, as opposed to the usual homogenous staining with a bright peripheral ring and occasional bright nucleoplasmic nucleoli. Foci were usually located on the nuclear periphery and were very bright patches of Lamin A/C staining.

Emerin foci were judged as bright staining patches, as opposed to the usual evenly distributed peripheral ring. Emerin immunosignal was too weak to analyse honeycomb mislocalization reliably. Lamin B1 capping was assessed as nuclei with regions of absent immunoreactivity, as opposed to its usual homogenous distribution with a slightly brighter peripheral ring.

For comparisons between 2D monolayer vs. 3D artificial skeletal muscle, the SPE2 confocal microscope was utilized to image randomly selected fields with an objective x63, zoom factor equal to 1.5 and a 0.5 μm gap between the slices (2D monolayer: between 82 and 154 nuclei were analyzed across 10–16 different randomly selected regions per cell line per repeat; 3D artifical muscle: between 37 and 70 nuclei were scored across 6–8 different randomly selected fields per cell line per repeat. Three repeats of independent cell passages were used). Three-dimensional reconstruction of nuclei was conducted with Imaris 8.4.1 software (www.bitplane.com). The 3D reconstructions were used for nuclear length measurements (conducted by measuring the major axis using Imaris 8.4.1 software) and shape classification (conducted as described above).

## Ethics and statistics

Work with human cells was performed under approval of the NHS Health Research Authority Research Ethics Committee reference no. 13/LO/1826; IRAS project ID no. 141100. GraphPad software Prism 6 was used for all statistical analysis and details of statistical tests are available in the Results section and in figure legends. Statistical significance was set with a *P* value of < 0.05.

## Author contributions

FST and PSZ: conceptualization, supervision, coordination, and funding; HBS-S with the help of LP, SS, TO, and SMM: methodology and investigation; HBS-S, LP, FST, and PSZ: data analysis and discussion; HBS-S, FST, and PSZ: wrote the manuscript.

### Conflict of interest statement

FST was principal investigator on a research grant from Takeda New Frontier Science Program (2014-2016), received speaking and consulting fees by Takeda and Sanofi-Genzyme (via UCL Consultants) and has a collaboration with GSK via a BBSRC iCASE studentship (BB/N503915/1; unrelated research project). The remaining author declares that the research was conducted in the absence of any commercial or financial relationships that could be construed as a potential conflict of interest. The handling Editor declared a shared affiliation, though no other collaboration, with several of the authors HBS-S, LP, TO, PSZ.

## Supplementary material

Supplementary Figure 1 Generation of three *LMNA*-mutant inducible myogenic cell lines from laminopathy patient-derived iPSCs. **(A)** FACS histograms showing immunophenotype of the K32del, R249W, and L35P *LMNA*-mutant inducible myogenic lines is similar to a previously-generated control line (Maffioletti et al., 2015). **(B)** Immunofluorescence showing absence of pluripotency-associated markers SOX2 and OCT3/4 in the differentiated cells (control iPSCs: positive control). **(C)** Immunofluorescence panel showing absence of Lamin A/C expression in a *LMNA*-mutant iPSC colony, but faint expression in the differentiating cells delaminating from the same colony. Scale bars: 100 μM.

Supplementary Figure 2 Lamin A/C localization to the nuclear periphery and nucleoplasm in K32del, R249W, and L35P HIDEMs and myotube nuclei. **(A)** Distribution of fluorescence intensity values across the nucleus (dotted line) in control and *LMNA*-mutant HIDEMs (K32del shown as a representative example). **(B)** Evaluation of peripheral Lamin A/C was calculated by dividing the average fluorescence intensity of the nuclear periphery (nuclear lamina; b) by the average fluorescence intensity of the nucleoplasm (a), generating the peripheral localization ratio. A ratio above 1 denotes more Lamin A/C is located at the nuclear periphery than the nucleoplasm. **(C)** Upper bar graph showing Lamin A/C peripheral localization ratio in *LMNA*-HIDEMs (only L35P has significantly less peripheral Lamin A/C in comparison to both controls). **(D)** Quantification of the proportion of cells with some degree of peripheral immunolabeling (peripheral/nucleoplasmic Lamin A/C > 1.05). Only L35P *LMNA*-mutant HIDEMs had significantly less peripheral Lamin A/C in comparisons to both control cell lines. **(E,F)** Analysis completed as for **(C,D)** in HIDEMs terminally differentiated into myotubes. Data show results from myonuclei. **(G,H)** Direct comparison of values in graphs **(C–F)** showing differences between peripheral localization ratios in HIDEMs and terminally differentiated myonuclei. Nuclear peripheral and nucleoplasmic regions were manually selected using the polygon tool in Fiji. Three passages were analyzed per cell line (*n* = 3), with 61-114 cells analyzed per passage per cell line. **(C,D)** One-way ANOVA with Tukey's *post-hoc* comparisons to control. **(E–H)** Two-way ANOVA with Tukey's *post hoc* comparisons to control, and Sidak's *post-hoc* for comparisons between HIDEMs and myonuclei. Data are shown as box a whiskers plots based on all values from three repeats combined (**C** and **G**), or the proportion of nuclei per imaging field (**D** and **H**), whiskers: min and max values, +: mean of all values plotted, numbers on graph show average of three repeats. ^*^*p* < 0.05, ^**^*p* < 0.01, ^***^*p* < 0.001, ^****^*p* < 0.0001.

Supplementary Figure 3 Lamin B1 and Emerin localization to the nuclear periphery and nucleoplasm in K32del, R249W and L35P HIDEMs. **(A)** Distribution of Lamin B1 fluorescence intensity values across the nucleus (dotted line) in control and *LMNA*-mutant HIDEMs (K32del shown as a representative example). **(B)** Evaluation of peripheral Lamin B1 was calculated by dividing the average fluorescence intensity of the nuclear periphery (nuclear lamina, b) by the average fluorescence intensity of the nucleoplasm (a), generating the peripheral localization ratio. A ratio above 1 denotes more Lamin B1 is located at the nuclear periphery than the nucleoplasm. **(C)** Upper bar graph showing Lamin B1 peripheral localization ratio in *LMNA*-HIDEMs. **(D)** Quantification of the proportion of cells with some degree of peripheral labeling (peripheral/nucleoplasmic Lamin B1 > 1.05). No *LMNA*-mutant HIDEMs had significantly less peripheral Lamin B1 for both comparisons to control cell lines. **(E–H)** Data and analyses as in **(A–D)** for Emerin. Nuclei peripheral and nucleoplasmic regions were manually selected using the polygon tool in Fiji. One-way analysis of co-variance (ANOVA), with Tukey's *post hoc* comparisons, ^*^*p* < 0.05, ^**^*p* < 0.01, ^***^*p* < 0.001. Data are shown as box a whiskers plots based on all values from three repeats combined **(C, E, G)**, or the proportion of nuclei per imaging field **(D, F, H)**, whiskers: min and max values, +: mean of all values plotted, numbers on graph show average of three repeats.

Supplementary Figure 4 *LMNA*-mutant iPSCs myotubes in 3D artificial skeletal muscle constructs. Representative maximum projections of confocally imaged human artificial muscle constructs of lines K32del, R249W and L35P immunolabeled for Lamin A/C and the marker of terminal myogenic differentiation Titin with all nuclei counterstained with Hoechst. Confocal stacks spanned the entire z-plane of the gels, each slice was 1 μm in thickness with a 0.5 μm overlap with the next slice. Scale bar: 30 μm.

Supplementary Video 1 Three dimensional computer-generated reconstruction of confocal immunofluorescence showing nuclear morphology (Lamin A/C in red) and myotubes (eMyHC in green) in a monolayer of healthy donor hiPSC-derived myotubes.

Supplementary Video 2 Three dimensional computer-generated reconstruction of confocal immunofluorescence showing nuclear morphology (Lamin A/C in red) and myotubes (eMyHC in green) in a monolayer of K32del *LMNA*-mutant hiPSC-derived myotubes.

Supplementary Video 3 Three dimensional computer-generated reconstruction of confocal immunofluorescence showing nuclear morphology (Lamin A/C in red) and myotubes (eMyHC in green) in a monolayer of L35P *LMNA*-mutant hiPSC-derived myotubes.

Supplementary Video 4 Three dimensional computer-generated reconstruction of confocal immunofluorescence showing nuclear morphology (Lamin A/C in red) and myotubes (eMyHC in green) in a monolayer of R249W *LMNA*-mutant hiPSC-derived myotubes.

Supplementary Video 5 Three dimensional computer-generated reconstruction of confocal immunofluorescence showing nuclear morphology (Lamin A/C in red) and myotubes (eMyHC in green) in a human artificial muscle derived from healthy donor hiPSCs.

Supplementary Video 6 Three dimensional computer-generated reconstruction of confocal immunofluorescence showing nuclear morphology (Lamin A/C in red) and myotubes (eMyHC in green) in a human 3D artificial muscle derived from K32del *LMNA*-mutant iPSCs.

Supplementary Video 7 Three dimensional computer-generated reconstruction of confocal immunofluorescence showing nuclear morphology (Lamin A/C in red) and myotubes (eMyHC in green) in a human 3D artificial muscle derived from L35P *LMNA*-mutant iPSCs.

Supplementary Video 8 Three dimensional computer-generated reconstruction of confocal immunofluorescence showing nuclear morphology (Lamin A/C in red) and myotubes (eMyHC in green) in a human 3D artificial muscle derived from R249W *LMNA*-mutant iPSCs.
