# Supplementary figures and images for: Modeling Skeletal Muscle Laminopathies Using Human Induced Pluripotent Stem Cells Carrying Pathogenic LMNA Mutations
Source: Front Physiol. 2018 Oct 15;9:1332. doi: 10.3389/fphys.2018.01332 (PMC6201196; doi:10.3389/fphys.2018.01332)

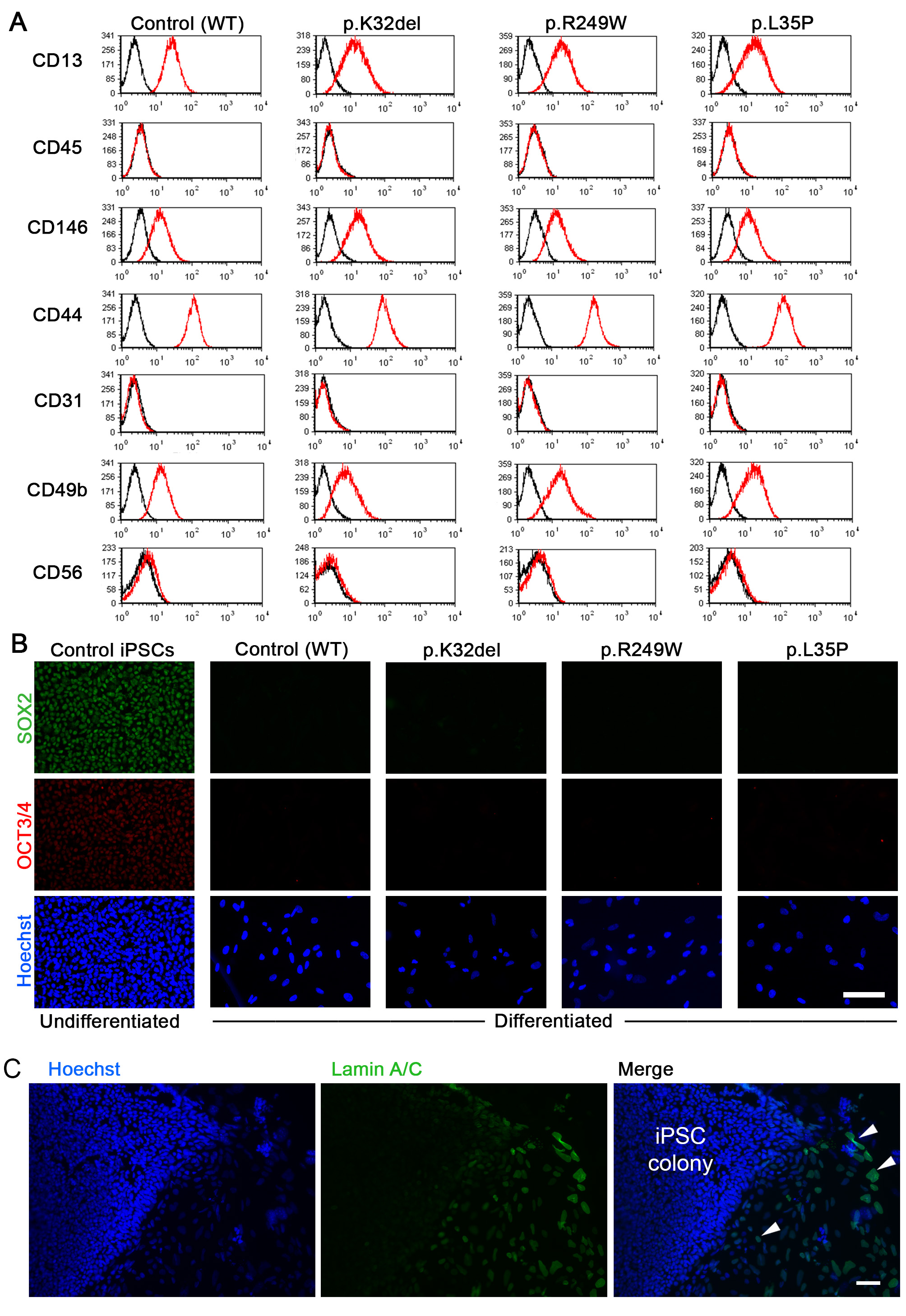

Supplement: Supplementary Figure 1 — Generation of three LMNA-mutant inducible myogenic cell lines from laminopathy patient-derived iPSCs. (A) FACS histograms showing immunophenotype of the K32del, R249W, and L35P LMNA-mutant inducible myogenic lines is similar to a previously-generated control line (Maffioletti et al., 2015). (B) Immunofluorescence showing absence of pluripotency-associated markers SOX2 and OCT3/4 in the differentiated cells (control iPSCs: positive control). (C) Immunofluorescence panel showing absence of Lamin A/C expression in a LMNA-mutant iPSC colony, but faint expression in the differentiating cells delaminating from the same colony. Scale bars: 100 μM. [file Image_1.JPEG]

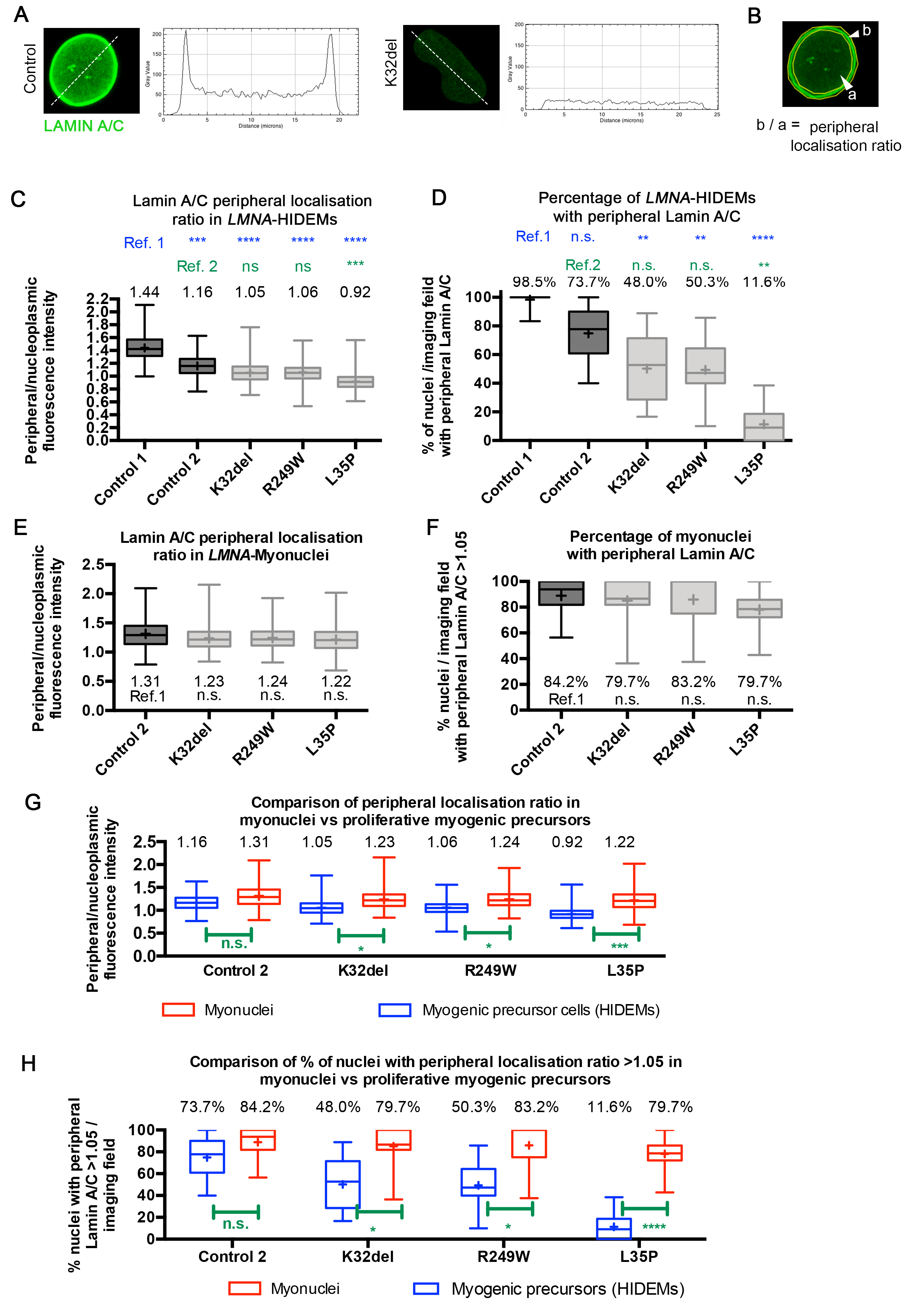

Supplement: Supplementary Figure 2 — Lamin A/C localization to the nuclear periphery and nucleoplasm in K32del, R249W, and L35P HIDEMs and myotube nuclei. (A) Distribution of fluorescence intensity values across the nucleus (dotted line) in control and LMNA-mutant HIDEMs (K32del shown as a representative example). (B) Evaluation of peripheral Lamin A/C was calculated by dividing the average fluorescence intensity of the nuclear periphery (nuclear lamina; b) by the average fluorescence intensity of the nucleoplasm (a), generating the peripheral localization ratio. A ratio above 1 denotes more Lamin A/C is located at the nuclear periphery than the nucleoplasm. (C) Upper bar graph showing Lamin A/C peripheral localization ratio in LMNA-HIDEMs (only L35P has significantly less peripheral Lamin A/C in comparison to both controls). (D) Quantification of the proportion of cells with some degree of peripheral immunolabeling (peripheral/nucleoplasmic Lamin A/C > 1.05). Only L35P LMNA-mutant HIDEMs had significantly less peripheral Lamin A/C in comparisons to both control cell lines. (E,F) Analysis completed as for (C,D) in HIDEMs terminally differentiated into myotubes. Data show results from myonuclei. (G,H) Direct comparison of values in graphs (C–F) showing differences between peripheral localization ratios in HIDEMs and terminally differentiated myonuclei. Nuclear peripheral and nucleoplasmic regions were manually selected using the polygon tool in Fiji. Three passages were analyzed per cell line (n = 3), with 61-114 cells analyzed per passage per cell line. (C,D) One-way ANOVA with Tukey's post-hoc comparisons to control. (E–H) Two-way ANOVA with Tukey's post hoc comparisons to control, and Sidak's post-hoc for comparisons between HIDEMs and myonuclei. Data are shown as box a whiskers plots based on all values from three repeats combined (C and G), or the proportion of nuclei per imaging field (D and H), whiskers: min and max values, +: mean of all values plotted, numbers on graph show average of three rep [file Image_2.JPEG]

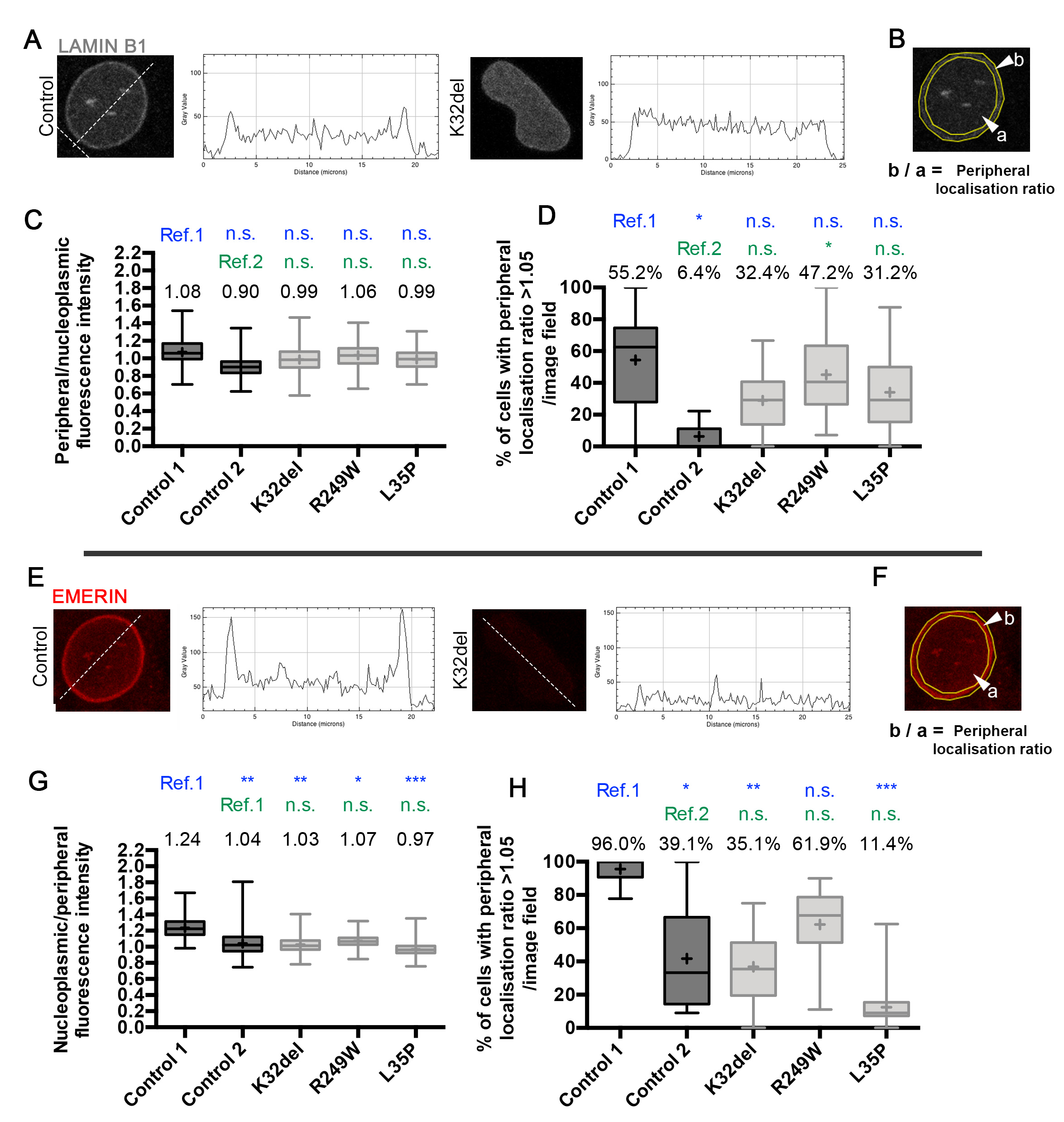

Supplement: Supplementary Figure 3 — Lamin B1 and Emerin localization to the nuclear periphery and nucleoplasm in K32del, R249W and L35P HIDEMs. (A) Distribution of Lamin B1 fluorescence intensity values across the nucleus (dotted line) in control and LMNA-mutant HIDEMs (K32del shown as a representative example). (B) Evaluation of peripheral Lamin B1 was calculated by dividing the average fluorescence intensity of the nuclear periphery (nuclear lamina, b) by the average fluorescence intensity of the nucleoplasm (a), generating the peripheral localization ratio. A ratio above 1 denotes more Lamin B1 is located at the nuclear periphery than the nucleoplasm. (C) Upper bar graph showing Lamin B1 peripheral localization ratio in LMNA-HIDEMs. (D) Quantification of the proportion of cells with some degree of peripheral labeling (peripheral/nucleoplasmic Lamin B1 > 1.05). No LMNA-mutant HIDEMs had significantly less peripheral Lamin B1 for both comparisons to control cell lines. (E–H) Data and analyses as in (A–D) for Emerin. Nuclei peripheral and nucleoplasmic regions were manually selected using the polygon tool in Fiji. One-way analysis of co-variance (ANOVA), with Tukey's post hoc comparisons, *p < 0.05, **p < 0.01, ***p < 0.001. Data are shown as box a whiskers plots based on all values from three repeats combined (C, E, G), or the proportion of nuclei per imaging field (D, F, H), whiskers: min and max values, +: mean of all values plotted, numbers on graph show average of three repeats. [file Image_3.JPEG]

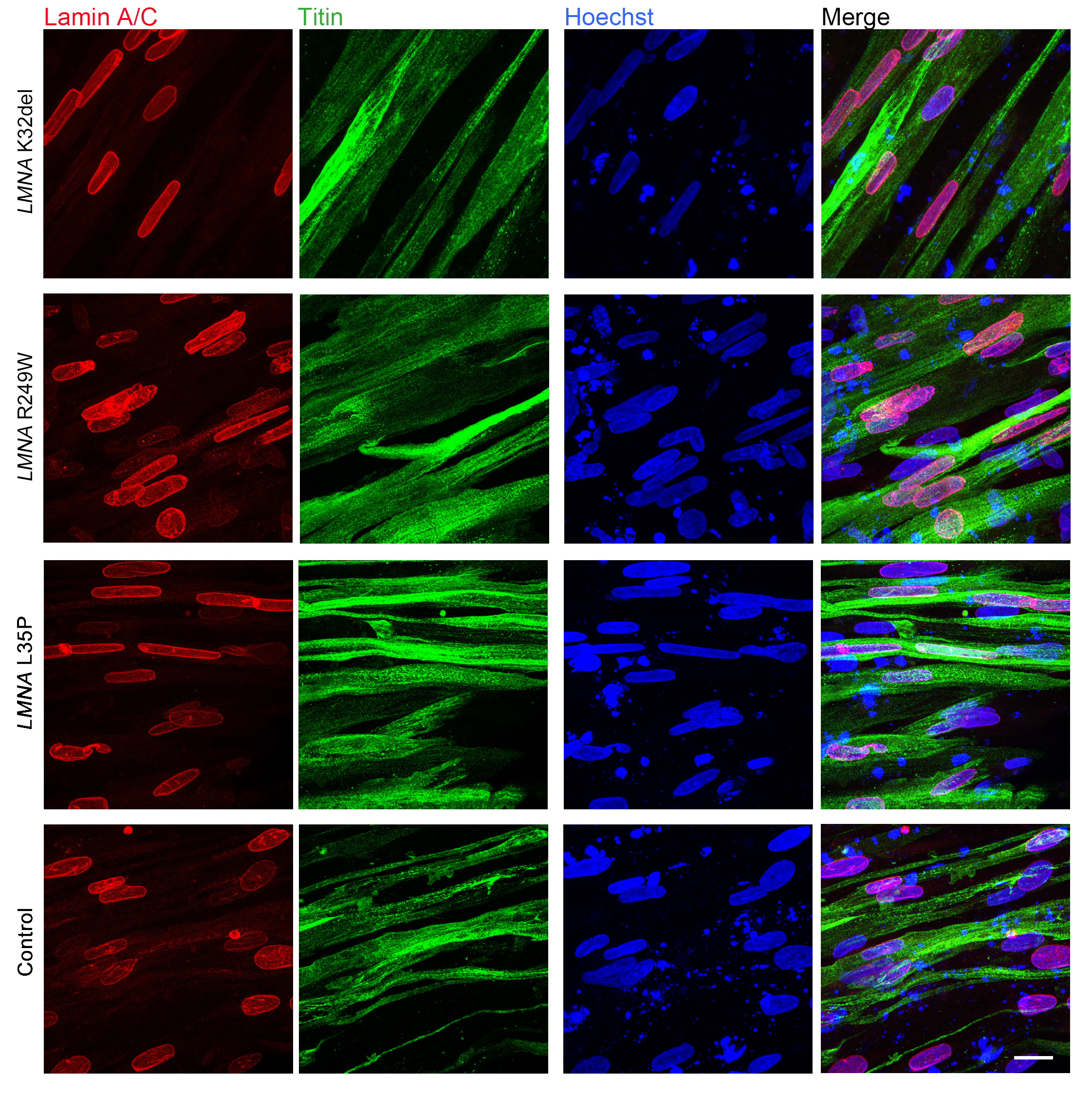

Supplement: Supplementary Figure 4 — LMNA-mutant iPSCs myotubes in 3D artificial skeletal muscle constructs. Representative maximum projections of confocally imaged human artificial muscle constructs of lines K32del, R249W and L35P immunolabeled for Lamin A/C and the marker of terminal myogenic differentiation Titin with all nuclei counterstained with Hoechst. Confocal stacks spanned the entire z-plane of the gels, each slice was 1 μm in thickness with a 0.5 μm overlap with the next slice. Scale bar: 30 μm. [file Image_4.JPEG]
